# A cross‐sectional survey exploring knowledge, beliefs and barriers to whole food plant‐based diets amongst registered dietitians in the United Kingdom and Ireland

**DOI:** 10.1111/jhn.13386

**Published:** 2024-11-03

**Authors:** Michael Metoudi, Alexander Bauer, Tanya Haffner, Shireen Kassam

**Affiliations:** ^1^ Plant‐Based Health Professionals UK London UK; ^2^ Institute for Prevention and Cancer Epidemiology, Faculty of Medicine and Medical Center University of Freiburg Freiburg im Breisgau Germany; ^3^ MyNutriWeb London UK; ^4^ King's College London University of Winchester Hampshire UK

**Keywords:** planetary health, plant‐based diet, registered dietitian, sustainability, sustainable diets, vegan

## Abstract

**Background:**

Plant‐based diets (PBDs) have gained popularity in recent years due to their co‐benefits for human and planetary health. Although clinical guidelines now recommend well‐balanced PBDs, it remains unclear how dietitians in the United Kingdom (UK) and Republic of Ireland (ROI) view such diets and their implementation in practice. This study aimed to evaluate the perspectives of registered dietitians (RDs) on the role of a whole food plant‐based diet (WFPBD) in clinical practice.

**Methods:**

A cross‐sectional online survey was conducted amongst RDs in the UK and ROI between September and November 2023.

**Results:**

A total of 335 RDs participated in the survey. Most RDs (76%) agree that a well‐planned WFPBD is suitable for all stages of life, with some (48%) expressing concerns over the risk of malnutrition and micronutrient deficiencies. Three‐quarters of RDs (75%) incorrectly thought that plant protein is incomplete, necessitating protein combining. A WFPBD was on average ranked fourth out of 14 dietary strategies for managing some of the leading non‐communicable diseases. Excluding dairy and eggs was the primary reason preventing RDs from personally adopting a WFPBD, whereas meal preparation and cost were the main barriers for their clients. The majority of RDs (79%) feel they did not receive sufficient education on WFPBDs, with 58% agreeing there are insufficient evidence‐based resources available in their field. Only 33% feel supported when advocating for a WFPBD in their workplace.

**Conclusions:**

Although many RDs view WFPBDs as viable and clinically relevant, significant barriers to their implementation exist, underscoring the need for enhanced education and support.

## Key points

- The majority of registered dietitians (RDs) from the UK and Republic of Ireland in this study hold a positive view of whole food plant‐based diets (WFPBDs) and are willing to recommend them to their patients.
- Considerable knowledge deficits related to current clinical evidence of WFPBDs exist amongst RDs, particularly, in the area of oncology. Additionally, three‐quarters of dietitians falsely believed that plant proteins are an incomplete source of protein.
- Malnutrition risk and micronutrient deficiencies, together with eating disorders appear to be a concern amongst many RDs for individuals transitioning to a WFPB dietary pattern.
- Limited education, a lack of a supportive work environment and excluding certain food groups remain considerable personal and professional barriers for RDs implementing WFPBDs in practice, whereas difficulties such as cooking skills and financial cost were the main perceived barriers for their patients in adopting a more plant‐based dietary pattern.
- These findings suggest a call to action amongst public health agencies and universities in providing further education and support to help RDs appropriately implement PBDs into their practice.

## INTRODUCTION

The surge in interest surrounding plant‐based diets (PBDs) in recent years has prompted a progressive shift in dietary patterns globally, owing to their positive impact on human health and environmental sustainability. Although there is no consensus definition on what constitutes a PBD, it does describe a dietary pattern that is centred around the consumption of predominantly plant foods while minimising or excluding animal‐based foods, akin to a vegetarian, vegan and/or flexitarian dietary pattern. However, it does not always consider the overall quality of the diet in terms of nutritional adequacy. A whole food plant‐based diet (WFPBD), first coined by Dr. T. Colin Campbell of Cornell University^1^ and later adopted by several public health agencies including the World Health Organization^2^ describes a diet that takes into account the overall nutritional quality and is a dietary approach that emphasises minimally processed, nutrient‐dense foods such as fruits, vegetables, whole grains, legumes, nuts and seeds while limiting animal foods.

Current evidence to date arising from a combination of observational and intervention studies has demonstrated the benefits that WFPB dietary patterns confer in reducing the risk of some of the world's commonest non‐communicable diseases.^3^, ^4^, ^5^, ^6^ They have also been shown to be one of the leading dietary patterns to exhibit the least impact on key environmental indicators for planetary health, including greenhouse gas emissions, land and water use and biodiversity loss, having the potential to offset the global impact that climate change has made in recent years.^7^, ^8^, ^9^, ^10^, ^11^, ^12^, ^13^ The 2019 report from the Eat‐*Lancet* Commission highlighted the need to transition to a predominantly PBD, not only for optimal health but to circumvent challenges faced amidst the current climate crises, laying out predefined future food sustainability goals by 2050.^14^ The planetary health diet, incepted from the report, describes a WFPBD to achieve these key milestones.

Several leading medical and nutritional institutions have now provided clear clinical guidance on PBDs, with position statements from dietetic institutions, including the Academy of Nutrition and Dietetics, Dietitians of Canada as well as the Dietitians Association of Australia now endorsing the suitability of a well‐planned PBD throughout all stages of life and for planetary health.^15^, ^16^, ^17^ Within the UK and Ireland, the British Dietetic Association, the National Health Service and the Irish Nutrition and Dietetic Institute (INDI) have also provided guidance on PBDs and their impact on human health.^18^, ^19^, ^20^ The BDA's One Blue Dot project, an initiative adopted in 2018, aimed at ensuring that dietary guidance is synonymous with healthy and sustainable eating.^21^ As such, there is a need for healthcare professionals (HCPs) to continue to adopt an awareness of the shift towards a more sustainable dietary pattern (SDP).

As nutritional professionals, RDs are considered experts at leading the way in nutritional interventions and are key stakeholders in terms of advocacy in public health nutrition and medical nutrition therapy. As such, they are strategically placed within healthcare in playing a critical role in reconciling sustainable food choice advice while meeting the nutritional needs of the population. It is not well known however to what extent RDs are implementing such dietary advice as part of standard dietetic care, particularly, for the UK and Republic of Ireland (ROI) and what their overall perspectives are on WFPBDs.

The aim of this research was to evaluate the perspectives of RDs in the UK and ROI on WFPBDs; specifically, their current knowledge, beliefs and attitudes and in what capacity they are implementing this dietary advice in the clinical setting. Evaluating such outcomes will help better understand and navigate the potential barriers dietitians may face when implementing these recommendations and whether there is a need for further education and support in this area.

## METHODS

### Survey and study design

A 35‐question cross‐sectional survey was developed by the first and last author in 2023. A literature search was first conducted to evaluate previous surveys of a similar nature, yielding limited results as this was a new area of research. Surveys by Krause and Williams^22^ evaluating medical physicians' understanding of WFPBDs and Hughes et al.^23^ amongst renal RDs provided a general basis for our survey. Results were reported in line with the Checklist for Reporting Results of Internet E‐Surveys criteria.^24^ Prior to data collection, one RD from the UK specialising in plant‐based nutrition and two RDs from the ROI and one nutritionist from the UK specialising in WFPBDs provided advice independently on the research study design and assisted in piloting the survey.

### Participant recruitment

The survey was distributed via email to members of MyNutriWeb (MNW), a UK web‐based nutritional and education organisation for HCPs, with approximately 24,500 members, subscribed members of the INDI, ROI, with approximately 1180 members, as well as being shared on social media. Only RDs practising within the UK and ROI were eligible to take part in the survey.

### Ethics and data protection

The study received ethical approval from the research ethics committee of the University of Winchester and was conducted according to current research ethical guidelines from the Declaration of Helsinki. Informed consent was obtained and documented from all participants at the beginning of the survey. Participant data was collected and stored on a secure and General data protection regulation (GDPR) compliant survey platform (available at: https://forms.office.com/), complying with Article 6(1)(e) of GDPR.

### Survey and data collection

The 35‐question survey comprised three sections: (i) participants' demographics, academic information and personal dietary habits, (ii) their current knowledge of plant‐based nutrition (e.g. nutrients of concern and their knowledge in the context of varying nutrition‐related health conditions) and (iii) the beliefs and attitudes on WFPBDs and their suitability in different clinical contexts (see Supporting Information S1: Section 4 for the full survey). Most of the survey was designed using a Likert scale and multiple‐choice items to generate knowledge and opinionated data in line with the study's aims. The survey remained active for a total of 12 weeks. Respondents were excluded on the basis of not being an RD, practising dietetics outside of the UK or ROI and the identification of corrupted data (e.g., obvious duplicates, inconsistent or invalid answers). A quality check was conducted by two reviewers independently (first and second author) to check for respondents with any inconsistencies in their responses to the survey. Based on this quality check, no respondents were excluded and only one age value (‘2 years’) was set to a missing value.

### Knowledge evaluation of WFPBDs amongst RDs

For the purpose of this study, a WFPBD was defined within the framework of the EAT‐*Lancet* planetary health diet, where ≥87% of total energy is derived from minimally processed whole plant foods (e.g., whole grains, fruits, vegetables, legumes) and where meat and dairy foods are minimised.^14^ This was used as the reference for each survey question related to a WFPBD. Knowledge of WFPBDs was evaluated based on three core knowledge domains related to the current clinical evidence: (i) the suitability of WFPBDs throughout the lifecycle based upon current evidence and clinical guidance, (ii) micronutrients of concern (both critical and non‐critical) following a WFPBD and (iii) current evidence of WFPBDs in managing or reducing the risk of certain chronic disease conditions (see Supporting Information S1: Figure S2). For each knowledge domain, an equal‐weighted knowledge score with values between 0 (all answers incorrect) and 1 (all answers correct) was calculated. RDs' knowledge related to plant proteins was assessed based on their understanding of whether they thought plant proteins to be incomplete (i.e., lacked certain essential amino acids).

### Statistical analysis

Potential differences in WFPB‐related knowledge, beliefs and barriers between RDs with specific subspecialties or more/less years of practice were evaluated descriptively. Inferential statistics were additionally applied to selected five knowledge dimensions (the role of WFPBDs within the life cycle, nutrients of concern on a WFPBD, nutrients of less or no concern on a WFPBD, evidence of WFPBDs in managing certain chronic diseases and quality of plant protein), three dimensions related to specific dietary strategies for managing type 2 diabetes (T2DM), cardiovascular disease (CVD) and weight management and two beliefs and attitudes dimensions (long‐term sustainability of WFPBDs and the frequency of recommending WFPBDs in clinical practice) to check for significant differences between these subspecialties, based on the estimation of 10 individual generalised linear regression models. Linear and logistic regression models were estimated for the knowledge domains and all other, binary variables, respectively. In each regression model, the independent variables comprised the subspecialty, the years of practice, the RDs' general level of education and the quality of RDs' education on WFPB diets (as a numeric score between 0 = ‘No WFPB‐specific education’ and 5 = ‘Excellent WFPB‐specific education’) as control variables. The WFPB recommendation model additionally consisted of (I) the existence of enough educational materials on PBDs and (II) the quality of support RDs receive in their respective places of work for WFPBDs advocacy as control variables. Age was not explicitly adjusted for since it strongly correlated with years of practice (linear correlation 0.80). No further relevant multicollinearity between independent variables or the potentially overlapping dietetic subspecialties was observed. Given that the area of focus is on comparing practising RDs, six RDs with a purely academic background were excluded from the data prior to model estimation. To ensure that the inclusion of ‘newly practising’ RDs (i.e., with less than 3 years of practical experience) does not bias the regression estimates, we re‐estimated all regression models as part of a sensitivity analysis while excluding all 75 newly practising RDs. Since no relevant changes in any model results were observed, we only report results based on the complete sample. A significance threshold of 0.05 was used. Full regression results can be found in Supporting Information S1: Tables S1–10. Model residuals were checked visually and did not show any substantial deviations from the model assumptions. For the logistic models, area under the curve (AUC) values were calculated on a randomly selected 20% hold‐out test set after re‐estimating each model on the remaining 80% training set. AUC values ranged between 0.56 and 0.68 (see Supporting Information S1: Tables S5–10).

Statistical analysis was performed using the open‐source software R.^25^ Regression models were estimated with function ‘gam’ from package ‘mgcv’,^26^ AUC values were calculated using function ‘calc_auc’ from package ‘plotROC’.^27^ Code and data for this study are accessible on GitHub.

### Qualitative analysis for open‐ended responses

Respondents' ‘additional thoughts and comments’ were evaluated using thematic analysis, a method for identifying and reporting patterns (themes) within data as previously described.^28^, ^29^ Open‐ended responses were evaluated by generating several inductive codes and keywords using MAXQDA Analytics Pro (24.4.0) software^30^ highlighted from the data. Emerging themes were then generated and evaluated from the total responses as previously described.^31^, ^32^

## RESULTS

### Participant demographics

A total of 471 responses were gathered between September and November 2023 (418 responders from MNW (2% response rate) and 53 responders from the INDI (4.5% response rate). Following a data quality check, a total of 335 were deemed eligible and used in the final analysis (Table 1). One hundred and thirty‐six respondents were excluded for not being an RD (*n* = 34), practising outside of the UK and ROI (*n* = 101) and one being a student (*n* = 1). 97% of participants identified as female. The median age of respondents was 38 years. 57% were educated to a postgraduate degree level. The majority of RDs (84%) worked within the hospital setting and/or primary care. Seven main specialty areas of dietetics were identified amongst respondents, including weight management and obesity (only referred to as ‘weight management’ in the following) (22%), diabetes (16%), gastroenterology (15%), paediatrics (14%), care for the elderly (14%), oncology (10%) and eating disorders (EDs) (9%) and were the main area of focus, representing 77% of RDs in this study.

**Table 1:** Baseline characteristics of dietitian participants in the UK and Ireland.

	Total population, *n* (%)	Omnivorous dietary pattern, *n* (%)		Semi‐vegetarian, *n* (%)	Vegetarian, *n* (%)	Plant‐based dietary pattern, *n* (%)		
Variable		Omnivore, *n* = 103 (31%)	Mediterranean, *n *= 56 (17%)	Flexitarian and pescetarian, *n* = 102 (30%)	Lacto and/or ovo vegetarian, *n* = 30 (9%)	WFPBD, *n* = 20 (6%)	Vegan, *n* = 24 (7%)	*p* Value^a^
Sex								
Female	324 (97)	97 (94)	53 (95)	101 (99)	30 (100)	19 (95)	24 (100)	
Male	10 (3)	5 (5)	3 (5)	1 (1)	0 (0)	1 (1)	0 (0)	
Prefer not to say	1 (1)	1 (100)	0 (0)	0 (0)	0 (0)	0 (0)	0 (0)	
Age^b^ (years)								0.0442*
20–29	73 (22)	20 (19)	9 (16)	24 (24)	9 (30)	4 (20)	7 (29)	
30–39	108 (32)	38 (37)	15 (27)	33 (32)	7 (23)	8 (40)	7 (29)	
40–49	87 (26)	14 (25)	14 (25)	28 (27)	8 (27)	4 (20)	10 (42)	
≥50	66 (20)	18 (32)	18 (32)	17 (17)	6 (20)	4 (20)	0 (0)	
Median age	38	38	42.5	36.5	36	37	35	
Level of education								0.4079
Bachelor's degree	142 (42)	45 (44)	22 (39)	45 (44)	11 (37)	9 (45)	10 (42)	
Postgraduate qualification	172 (51)	48 (47)	34 (61)	49 (48)	17 (57)	11 (55)	13 (54)	
PhD	20 (6)	10 (10)	0 (0)	8 (8)	1 (<1)	0 (0)	1 (4)	
Years working as a dietitian (years)								0.0880
≤3	75 (22)	25 (24)	11 (20)	21 (21)	7 (22)	2 (10)	9 (38)	
4–6	63 (19)	18 (17)	8 (14)	19 (19)	6 (20)	8 (40)	4 (17)	
7–9	34 (10)	12 (12)	5 (9)	7 (7)	3 (10)	2 (10)	5 (21)	
10–14	52 (16)	16 (16)	10 (18)	18 (18)	0 (0)	4 (20)	4 (17)	
15–19	34 (10)	8 (8)	6 (11)	13 (13)	4 (13)	1 (5)	2 (8)	
20–24	29 (9)	9 (9)	7 (12)	10 (10)	3 (10)	0 (0)	0 (0)	
≥25	48 (14)	15 (15)	9 (16)	14 (14)	7 (23)	3 (15)	0 (0)	
Median years of practice	9	8	11.5	10.5	8	6.5	5.5	
Area of work								0.0560
Hospital	148 (40)	47 (40)	18 (32)	54 (48)	9 (27)	8 (35)	12 (41)	
Primary care/community	135 (36)	44 (37)	29 (52)	35 (31)	10 (30)	8 (35)	9 (31)	
Private practice	42 (11)	7 (6)	5 (9)	11 (10)	7 (21)	6 (26)	6 (21)	
Academia/research	25 (7)	12 (10)	2 (4)	7 (6)	3 (9)	0 (0)	1 (3)	
Public health	22(7)	8 (7)	2 (4)	6 (5)	4 (12)	1 (4)	1 (31)	
Selected areas of specialty^c^								0.5155
Weight management	83 (22)	20 (17)	18 (26)	22 (22)	8 (29)	8 (28)	7 (24)	
Diabetes	59 (16)	16 (14)	8 (12)	19 (19)	6 (21)	8 (28)	2 (7)	
Gastroenterology	57 (15)	20 (17)	7 (10)	16 (16)	6 (21)	4 (14)	4 (14)	
Paediatrics	54 (14)	18 (15)	8 (12)	17 (17)	5 (18)	1 (3)	5 (17)	
Care for the elderly	51 (14)	18 (15)	9 (13)	11 (11)	2 (7)	5 (17)	6 (21)	
Oncology	36 (10)	15 (13)	10 (14)	9 (9)	0 (0)	1 (3)	1 (3)	
Eating disorders	35 (19)	11 (3)	9 (13)	8 (8)	1 (4)	2 (7)	4 (14)	

*Note*: Baseline characteristics of total participants (*n* = 335), MNW (*n* = 282) and INDI (*n* = 53).

Abbreviations: ANOVA, analysis of variance; INDI, Irish Nutrition and Dietetic Institute; MNW, MyNutriWeb.

^a^ ANOVA (for metric age and ‘years working as a dietitian’) or *χ* ^2^ test of independence‐based *p* value testing for a potential association between each listed variable and the dietitians' personal dietary pattern; significance threshold <0.05.

b Omission of one participant's age as imputed as ‘2 years’ in the survey.

^c^ The question related to the area of specialty was multiple choice. Additional RD subspecialties that were less commonly represented are not listed in the table. The subspecialties listed in the table represent 77% of the total RD respondents.

* Statistically significant.

### Evaluation of a PBD pattern amongst RDs

RDs' understanding of what constitutes a PBD was assessed from a list of common dietary patterns. The majority of RDs (63%) considered a vegan diet to most resemble a PBD pattern (Supporting Information S1: Figure S1A). The commonest personal dietary habits (Table 1, Supporting Information S1: Figure S1B) amongst participants were an omnivorous dietary pattern (46%) followed by a flexitarian dietary pattern (30%). Those who followed a vegan diet (7%) were shown to be significantly younger in age, relative to the other personal dietary groups, with a median age of 35 years (*p* = 0.0442) (Table 1). When assessing RDs' overall dietary patterns, 65% said that they had personally tried transitioning to a WFPBD, as defined as the EAT‐*Lancet* dietary pattern (Supporting Information S1: Figure S1C).

### Knowledge of WFPBDs in the clinical setting

**Figure 1:**
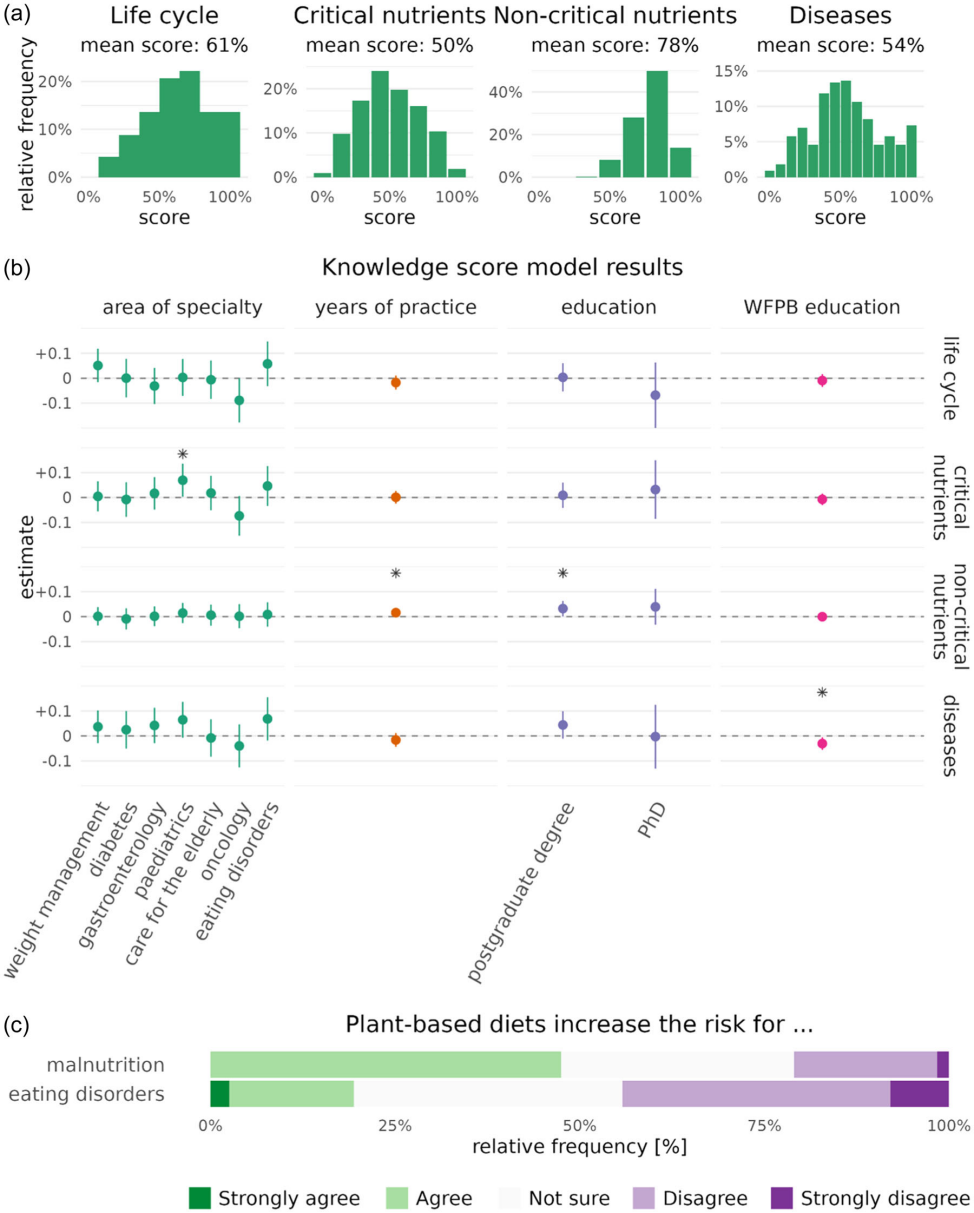
Knowledge of a whole food plant‐based diet (WFPBD) amongst registered dietitians. (a) showing the distribution of RDs on their overall knowledge of WFPBDs; evaluated based on the three core knowledge domains related to (i) WFPBDs throughout the lifecycle stages (Domain 1), (ii) nutrients of concern when following a WFPBD (critical and noncritical, Domain 2A and 2B) and (iii) current clinical evidence of WFPBDs in the management of chronic lifestyle‐related diseases (Domain 3), with a maximum score of 100%. (b) Linear regression model estimates showing mean effects on each mean knowledge score, *Statistically significant effects (*p* < 0.05). Adjusted for years of practice, education and education in WFPB nutrition (c) Showing participant's views on whether WFPBDs increase the risk of malnutrition and/or eating disorders.

Figure 1a displays the knowledge scores' distribution amongst RDs for each knowledge domain. Approximately 38% of RDs scored 75% or more with knowledge related to WFPBDs in the life cycle (Domain 1), 12% scored above 75% regarding micronutrients of concern (Domain 2A), 64% scored above 75% for nutrients of less or no concern (‘noncritical nutrients’, Domain 2B) and 22% of RDs achieved a score of 75% and greater for knowledge related to WFPBDs' role in managing certain chronic diseases (Domain 3). Linear regression analysis (Figure 1b) further revealed respondents that tended to score the highest across all domains were RD's specialising in weight management and paediatrics (*p* = 0.0411, for Domain 2A), whereas those working in oncology had overall lower knowledge scores (*p* = 0.05, for Domain 1) on current clinical evidence of plant‐based nutrition (see Supporting Information S1: Tables S1–4). RDs were asked to identify what conditions a WFPB dietary pattern has been shown to reduce the risk of. Heart disease (94%), elevated cholesterol (91%), hypertension (82%) and T2DM (74%) were the most common chronic conditions RDs reported (Figure). The commonest micronutrients of concern that RDs reported when following a WFPBD were vitamin B12(90%), iron (82%) and calcium (70%) (Supporting Information S1: Figure S4).

When evaluating the nutritional profile of WFPBDs as a whole, 48% had concerns related to a WFPBD increasing the risk of malnutrition, with 21% of RDs believing it could have an effect on increasing the risk of an ED, whereas 30% remained unsure (Figure 1c).

### Plant proteins and their nutritional sufficiency

**Figure 2:**
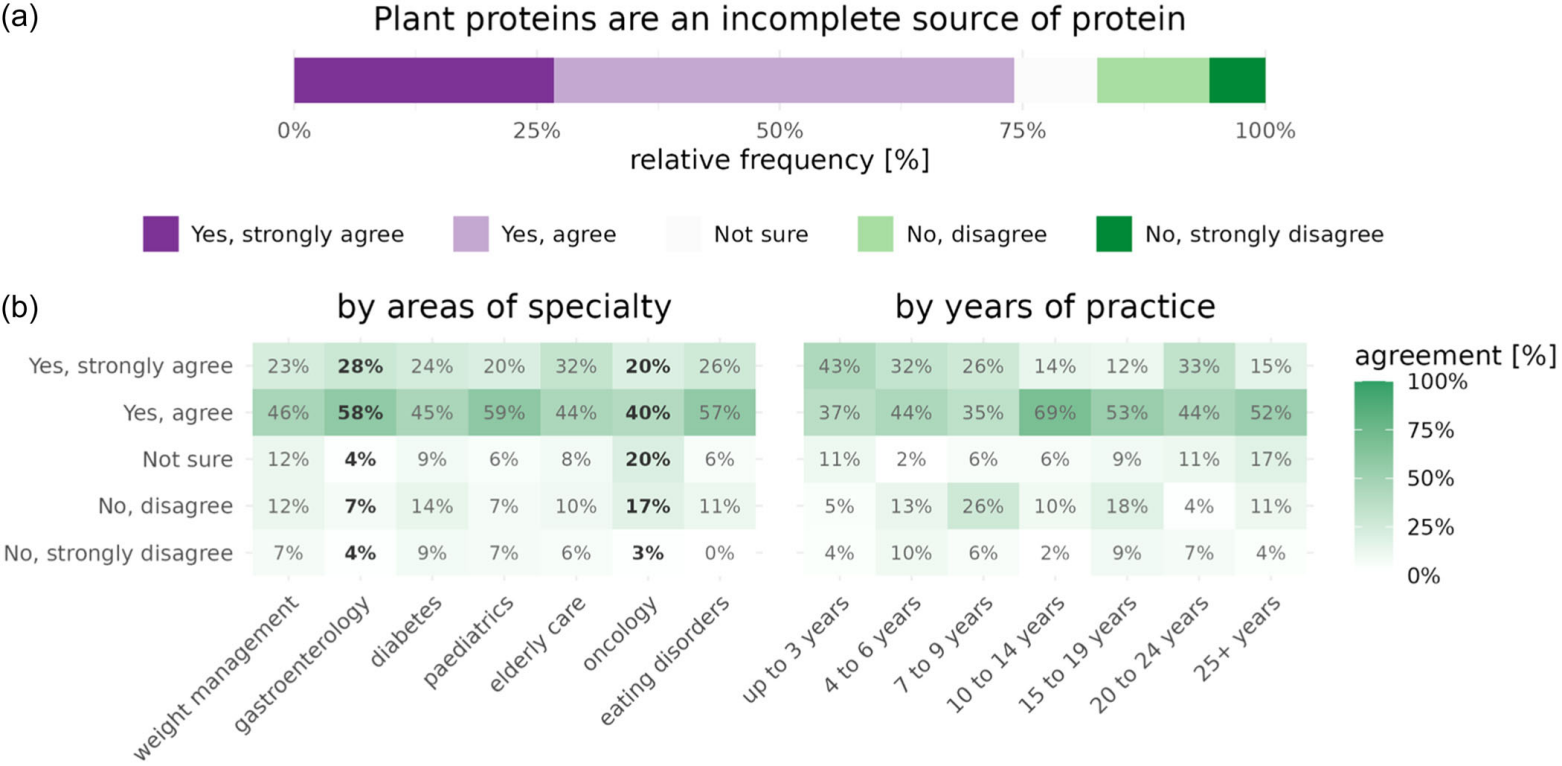
Plant protein sufficiency in a whole food plant‐based (WFPB) diet. (a) Showing registered dietitian's understanding of plant protein's nutritional adequacy when asked whether plant proteins are incomplete (lack certain essential amino acids and should be paired with other sources of plant protein). (b) Heat map showing participant responses according to specialty area and years of practice. Controlled for age, education, years of practice and education in WFPB nutrition. Percentages highlighted in bold reached statistical significance (*p* < 0.05).

Three‐quarters of dietitians (75%) across all specialties thought plant‐derived proteins were incomplete and should be carefully paired with other plant‐based protein foods for optimum protein requirements (Figure 2a). RD subgroups that significantly deviated from this overall knowledge pattern were RDs working in the fields of (i) oncology, of whom only 61% viewed plant‐based protein as incomplete (*p* = 0.0305) and (ii) gastroenterology, of whom 86% viewed plant‐based protein as incomplete (*p* = 0.0282) (Figure 2b, Supporting Information S1: Table S5).

### Dietary approaches for the management of chronic disease

**Figure 3:**
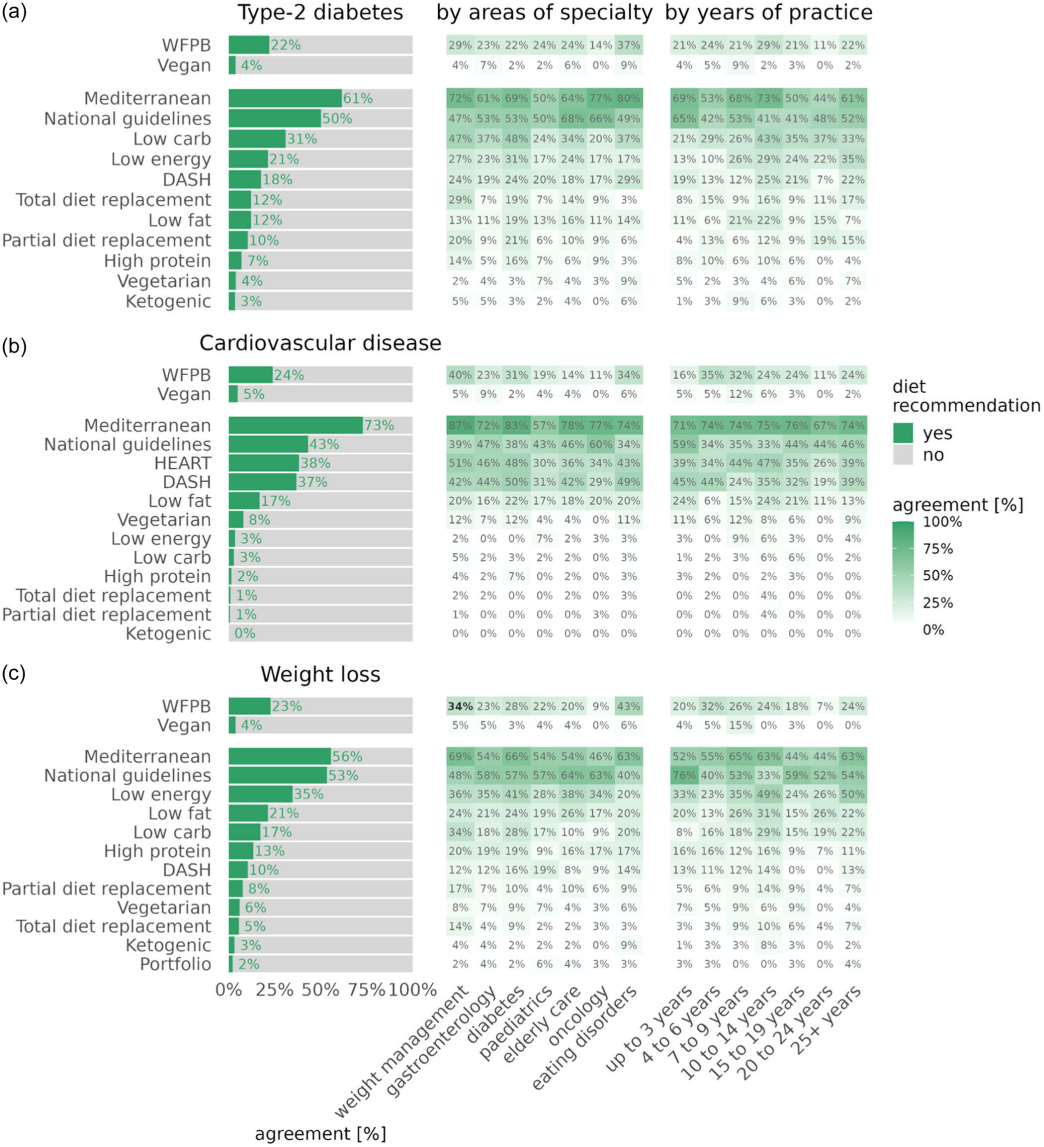
Dietary approaches for chronic disease management. Showing ranked plots and heat maps highlighting the preferred dietary management and prevention amongst registered dietitians for (a) type 2 diabetes (b) cardiovascular disease and (c) weight loss according to (i) area of specialty and (ii) years practising as a dietitian. Controlled for age, years of practice, education and education received on whole food plant‐based nutrition. Percentages highlighted in bold reached statistical significance (*p* ≤ 0.05).

RDs were asked to rate their preferred dietary approach in the management of some of the leading chronic, lifestyle‐related diseases. A Mediterranean dietary pattern was ranked as the primary intervention for managing T2DM and CVD as two areas of chronic disease, as well as for weight loss management as an additional dimension (Figure 3). A WFPBD ranked four out of 14 as the preferred dietary intervention for T2DM and weight loss and fifth for CVD, with 22%, 24% and 22% of RDs viewing a WFPBD as a viable and clinically relevant dietary pattern for managing T2DM, CVD and weight loss, respectively. This dietary pattern was rated especially positively by RDs working in the specific areas of diabetes and weight management. There appeared to be a trend for RDs working in weight management, who tended to choose WFPBDs as a recommended diet for the management of T2DM (although this did not reach statistical significance, 29% of RDs in this specialty area recommend this dietary pattern, *p* = 0.0993), CVD (40%, *p* ≤ 0.0001) and weight loss (34%, *p* = 0.0172), relative to the other specialty areas of dietetics (Supporting Information S1: Table S6–8).RDs specialising in EDs appeared to be more likely to recommend WFPBDs for T2DM (*p* = 0.0238) and weight loss (*p* = 0.0024). There also appeared to be a trend in oncology RDs, who were less likely to recommend WFPBDs for chronic disease management, including T2DM (14%, *p* = 0.3509), CVD (11%, *p* = 0.1142) and weight loss management (8%, *p* = 0.07), although this did not reach statistical significance. Regarding education, RDs with a postgraduate qualification were more likely to recommend WFPBDs in clinical practice for T2DM (+82% chance of recommendation compared to RDs with only an undergraduate degree, confidence interval (CI): [+3%, +221%], *p* = 0.0363) and CVD (+145%, CI: [+37%, +339%], *p* = 0.002) (Supporting Information S1: Tables S6–8).

### RDs' beliefs and attitudes of WFPBDs in clinical practice

**Figure 4:**
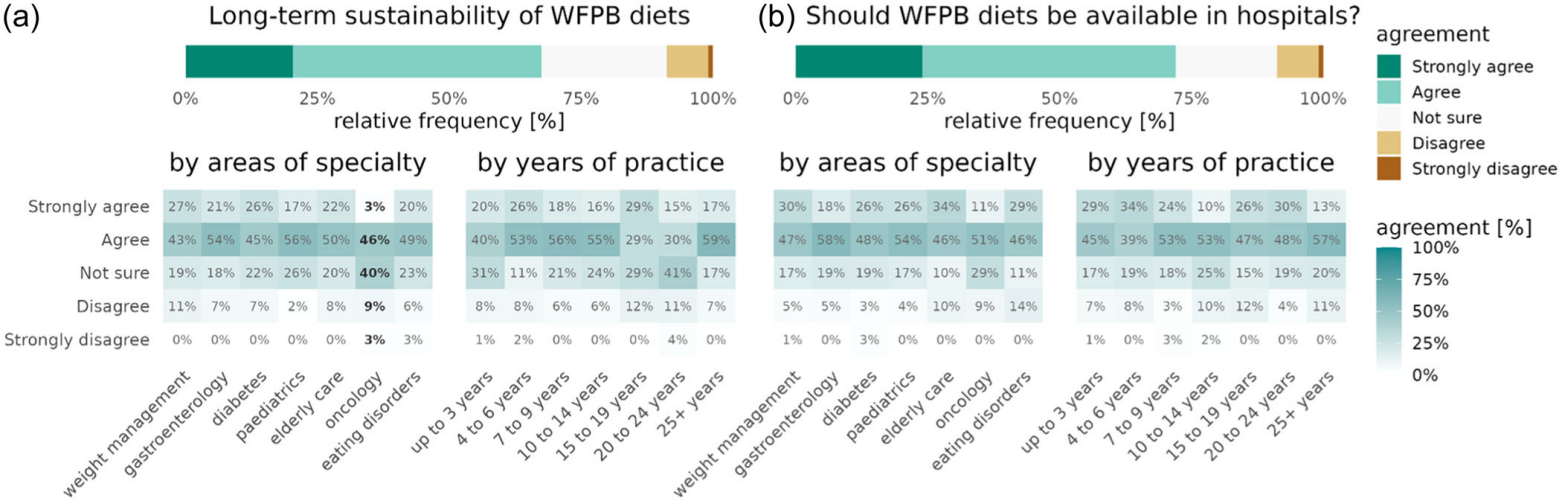
Registered dietitians' (RDs) beliefs attitudes on whole food plant‐baseds (WFPBDs) in clinical practice. Likert and heat map showing (a) RDs' opinions on whether adopting a WFPBD in being sustainable long‐term based on specialty area and years of practice as a dietitian. (b) RDs' beliefs on whether they thought WFPBDs should be integrated into hospitals and other healthcare settings for patients. Percentages highlighted in bold reached statistical significance (*p* ≤ 0.05).

Figure 4 highlights the overall core beliefs amongst RDs related to WFPBDs in clinical practice. 67% of RDs believed a WFPB dietary pattern to be sustainable long‐term (Figure 4a), whereas only 16% agreed that their clients with co‐morbidities would adhere long‐term to a WFPBD (Supporting Information S1: Figure S8). RDs working in oncology were significantly less likely to believe WFPBDs are generally sustainable long‐term (3%, *p* = 0.0011) compared to RDs working in other areas of dietetics. A trend was observed where oncology RDs were also the least likely to regularly recommend a WFPBD in their practice, although this did not reach statistical significance (3% always/often recommend WFPBDs, *p* = 0.1205, Supporting Information S1: Figure S5 and Table S10), whereas RDs working in EDs and weight management appeared to be the most likely to regularly recommend WFPBDs to their clients (*p* = 0.0024 and *p* = 0.0426). Those with a postgraduate degree were more likely to consider WFPBDs to be sustainable long‐term (+77% chance of agreement compared to undergraduate degrees, CI: [+5%, +183%], *p* = 0.0302, Supporting Information S1: Table S9) and tended to recommend WFPBDs in clinical practice more (+145%, CI: [+47%, +309%], *p* = 0.0004, Supporting Information S1: Table S10). The majority of RDs (70%) agreed that WFPBDs should be integrated and included as a recognised therapeutic dietary option in hospitals and other healthcare settings for appropriate patients. This was true irrespective of the specialty area (Figure 4b). When exploring the perceived motivations of their clients for transitioning to a WFPBD, most RDs said it was related to the environment (62%) and improving their overall health (58%) (Supporting Information S1: Figure S6).

### Barriers to implementing WFPBDs in clinical practice

**Figure 5:**
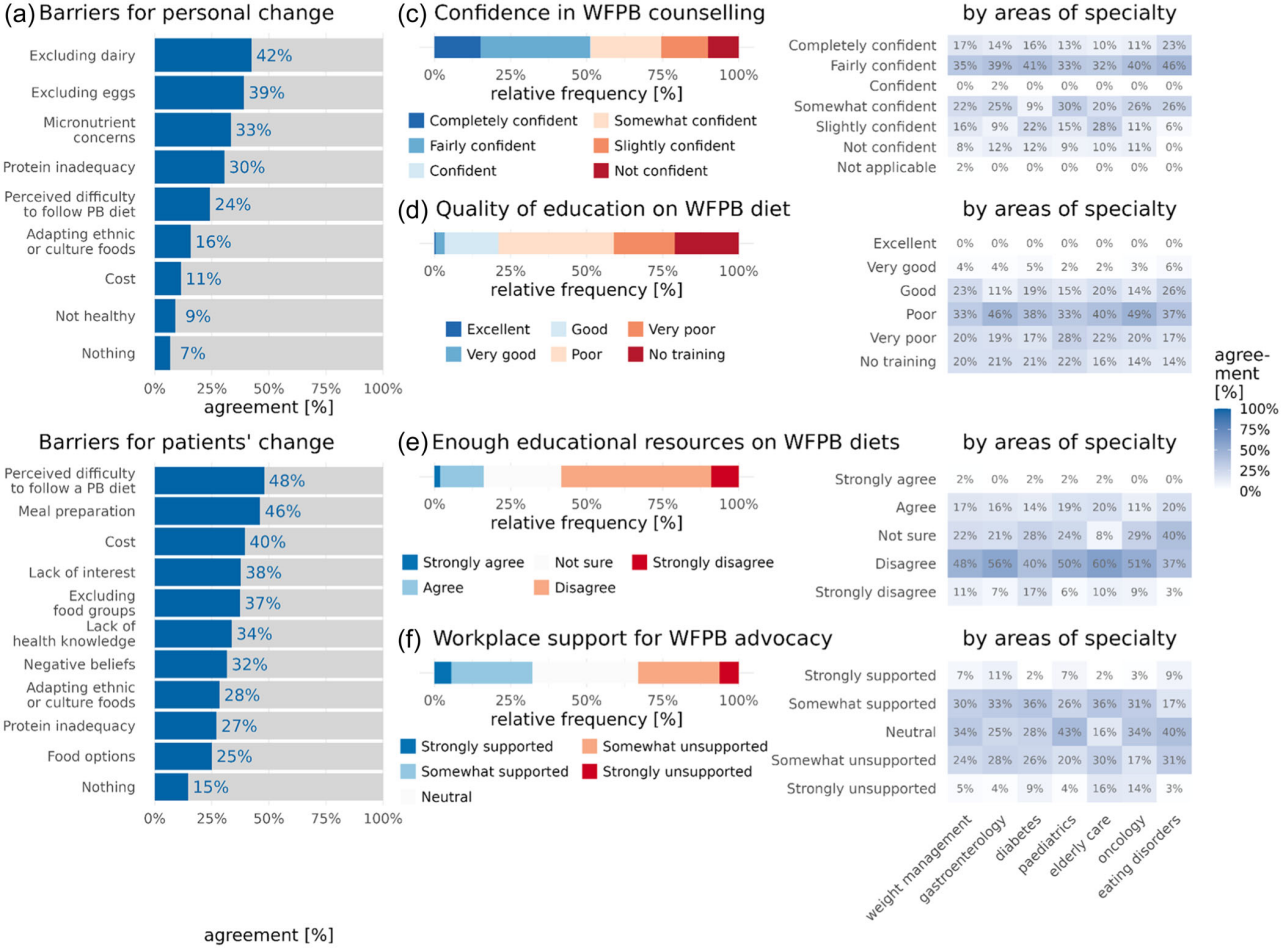
Barriers to implementing WFPBDs. (a) Personal barriers of registered dietitians (RDs) switching to a whole food plant‐based (WFPBD). (b) Main barriers clients and patients have in adopting a WFPBD. (c) RDs' overall confidence in counselling individuals to follow a WFPBD. (d) RDs' reflections on the adequacy of WFPB nutrition education and training in university and (e) adequacy in the availability of WFPB nutrition educational resources. (f) RDs' thoughts on whether they feel supported if advocating for a WFPB dietary pattern in the workplace.

Several barriers emerged for both RDs and their clients when exploring certain challenges if and when switching to a fully WFPB dietary pattern. Excluding dairy (42%) and eggs (38%), concerns over micronutrient intake (33%) and protein inadequacy (31%) were the primary reasons preventing RDs from fully adopting a WFPBD (Figure 5a). Concerns related to meal preparation (46%) and financial cost (39%) were revealed as the commonest perceived barriers for their clients, though only slightly more common compared to a considerable set of other barriers (Figure 5b).

Approximately 50% of RDs felt they were confident in counselling patients to switch to a WFPBD (Figure 5c). However, only 20% of RDs felt they received adequate training in plant‐based nutrition during their undergraduate dietetic degree, whereas 20% of all respondents declared that they received no training within the university (Figure 5d). 58% of RDs felt there were not enough evidence‐based educational resources for them and their patients to implement a WFPBD in practice (Figure 5e). This was particularly evident in those working in care for the elderly (69% practising in this specialty area).

When asked how regularly RDs recommend a WFPBD in their area of work, only 13% of dietitians would recommend WFPBDs in their respective specialties on a regular basis (Supporting Information S1: Figure S5). Only 33% of RDs feel supported if and when advocating for a WFPBD for their patients in their respective places of work. Further analysis revealed a substantially greater chance of RDs recommending a WFPBD the more they felt supported for doing so in the workplace (+39% for every unit increase on the 1–5 workplace support Likert scale, CI: [+8%, +79%], *p* = 0.0093) (Supporting Information S1: Figure S5 and Table S10). Approximately 27% of RDs did not believe that WFPBDs were realistic for individuals of lower socioeconomic status (SES), whereas 33% of RDs remained unsure (Supporting Information S1: Figure S7).

### Qualitative review of RDs' attitudes towards WFPBDs

A total of 96 RDs (25%) responded to the open‐ended textbox question, 86 (90%) of which were used, the rest were excluded for responses of ‘no comment’ or ‘N/A’. The majority of RDs who responded worked within the primary care setting (53%) with a median age of 38 years (Supporting Information S1: Table S11).

Seven core themes emerged from RDs following a comprehensive analysis of responses (Supporting Information S1: Figure S9 and Tables S12 and S13). The commonest theme (40%) was related to perceived barriers patients face for the practical implementation of WFPBDs (e.g., cooking skills, availability of plant‐based options and overall lack of interest) and the need for practical and realistic approaches for dietary changes, given what RDs believe to be the challenges clients already face in adhering to national dietary guidelines. Another main theme (24%) was RDs emphasising the importance of dietetic support for their clients in order to transition to a more WFPB dietary pattern while there still remains a significant lack of knowledge, education and support for patients and their healthcare providers in WFPB nutrition, emphasising a call for more educational and practical resources to support plant‐based eating. Other common themes were related to the importance of dietetic care remaining patient‐centred (15%) (understanding individualised patient needs, which is often context‐specific, and how WFPBDs could realistically be integrated as such). Further themes included nutrition and health concerns (15%) (nutritional adequacy and impact of excluding certain food groups), PBDs in EDs (11%) (concerns around the potential for restrictive eating behaviours used as a mask for EDs), socioeconomic themes (11%) (cost and affordability of WFPBDs) and public health and policy (7%) (the role of policies and institutions to promote and support plant‐based focused dietary changes).

## DISCUSSION AND CONCLUSION

To our knowledge, the present study is the first to explore the knowledge, beliefs and barriers concerning WFPBDs amongst RDs working across multiple clinical areas in the UK and ROI. The overarching findings of this study suggest that although RDs generally hold positive views of WFPBDs and acknowledge their health benefits, significant knowledge gaps and perceived barriers appear to prevent their broader integration into clinical practice. Our results are similar to prior published studies. Martinez et al,^33^ who evaluated the perspectives of SDPs amongst a cohort of European RDs, revealed a relatively larger proportion of RDs (38%) integrating SDP principles into their practice, yet similar primary barriers being a lack of training and overall structural support in the workplace existed. Opinions amongst a cohort of KwaZulu‐Natal RDs from South Africa,^34^ showed significant barriers to prescribing WFPBDs. Although the majority of these RDs acknowledged the health benefits of WFPBDs, they expressed concerns over a perceived difficulty in following a WFPBD, a lack of training in universities and concerns relating to suitability for lower‐income households. Standord et al.^35^ explored the perceptions of PBDs amongst a group of Australian renal RDs, who expressed similar barriers, such as lack of cooking and preparation skills, as well as financial cost. Finally, findings from Betz et al.^36^ amongst renal professionals within the United States, including RDs, similarly found that the majority of RDs believed PBDs could improve chronic diseases, such as chronic kidney disease, CVD and T2DM, with the primary barriers being perceived low acceptance from patients, higher cost and lack of workplace support.

Our findings similarly identify such core barriers. Although it appeared that most RDs in our study have tried transitioning to a more WFPB dietary pattern, there remained a personal and professional apprehension with excluding certain food groups (e.g., dairy and eggs) from the diet and concerns over micronutrient deficiencies being the main barriers, despite previous UK‐based studies suggesting reducing such foods is unlikely to compromise nutrient intake.^37^ Additionally, one of the most notable barriers highlighted amongst respondents was the perceived lack of education and resources available to practising RDs and during their training in university. This was evident even when stratifying for years of practice, suggesting education and training on plant‐based nutrition has not improved over time. It was also apparent that considerable barriers related to a lack of support for most RDs remain within the work setting. Interestingly, further exploration highlighted that RDs were more likely to recommend a WFPBD in their workplace if they felt supported in doing so, suggesting there to be an element of resistance within the work environment as an additional obstacle for RDs, aside from the barriers related to their patients. Financial cost and cooking abilities appeared to be the commonest perceived barriers for the RDs' patients, reflecting findings from the current literature previously mentioned. The commonality between the abovementioned studies, together with our findings, suggests a significant global issue amongst RDs and their perceptions towards PBDs in practice. We hypothesise several reasons for these findings, including: (I) cultural food norms and preferences, with traditional diets in these regions prioritising animal foods as primary protein sources, particularly, within the UK and ROI, where conservative dietary guidelines have emphasised a balanced diet to include animal foods. (II) A perception of higher costs for WFPB foods may exist in these regions, particularly if it involves fresh or specialty plant‐based food, despite such dietary patterns being generally more affordable.^38^, ^39^ (III) Finally, a lack of workplace support may be due to many country‐based dietary guidelines still emphasising animal‐source foods, as well as a lack of training and education leading to misconceptions amongst HCP colleagues on WFPB nutrition. This may be creating an unsupportive environment for RDs to advocate for a more PB dietary pattern.

Our survey reveals new insights, that while a considerable portion of RDs possess a good understanding of the benefits of WFPBDs in managing chronic diseases, such as CVD and T2DM, knowledge deficits are notable, particularly amongst oncology dietitians. This subgroup demonstrated lower knowledge scores on questions related to the clinical evidence supporting WFPBDs. We hypothesise this to be partly due to PBDs being less utilised in this area. Given the higher incidence of malnutrition in cancer patients,^40^ this often requires focusing on additional nutrition support measures during treatment, which traditionally, have been considered to be less plant‐based (e.g., prioritising energy‐dense animal‐based foods, food fortification practices, oral nutrition products, etc.) in order to circumvent conditions such as cancer cachexia and sarcopenia.^41^ Overall, this suggests a potential need for targeted educational initiatives and access to further evidence‐based resources to enhance their overall knowledge, utilising WFPBDs in a broader clinical context, given their emerging beneficial role in cancer management and prognoses.^42^, ^43^, ^44^, ^45^, ^46^ RDs working in weight management and paediatrics exhibited higher knowledge scores across all domains, indicating a more comprehensive understanding of the current clinical evidence of plant‐based nutrition. This disparity between disciplines further highlights the need for specialty‐specific training to ensure that all dietitians, regardless of their clinical area, are equally informed regarding the clinical and environmental evidence of WFPBDs.

Despite recognising the benefits, many RDs expressed concerns regarding the nutritional adequacy of WFPBDs. Although the majority considered key nutrients associated with WFPBDs (e.g., Vitamin B12, calcium and long‐chain omega‐3 fats Eicosapentaenoic acid (EPA) and Docosahexaenoic acid (DHA), iron intake was ranked as one of the leading nutrients of concern (i.e., risk of deficiency) across specialty areas, despite current evidence suggesting there to be no significant increased risk of iron deficiency amongst individuals following a WFPB dietary pattern.^47^, ^48^, ^49^ These results may however reflect findings from Beal et al.,^50^ who identified the potential for iron insufficiency in the EAT‐*Lancet* planetary health diet during pregnancy, warranting further evaluation of these findings. RDs' concerns over WFPBDs increasing the risk of malnutrition were noteworthy. This may in part be explained by further open‐ended responses revealing RDs' perceived concerns related to a WFPBD oftentimes being poorly planned, restricting certain food groups and individuals not sufficiently supplementing for key micronutrients. Despite a recent systematic review showing no increased risk of nutrient deficiencies in adults following a well‐planned PBD pattern compared to omnivorous diets,^48^ this highlights the need for a more nuanced approach in assessing which specific group RDs believe malnutrition to be a cause for concern in (e.g., toddlers, adolescents, older adults, those with poor appetites and at risk of malnutrition, etc). RDs' concerns, albeit less, also included the risk of EDs. However, data related to this area is very limited, with some results indicating no overt risks of ED behaviours amongst individuals following PBDs,^51^, ^52^ and perhaps some positive benefits.^53^, ^54^ However, this is a key area of focus requiring further research.

Additionally, the belief that plant proteins are incomplete and require careful pairing was prevalent, indicating that understanding of the protein quality of plants is lacking amongst a high percentage of respondents and still remains a widely accepted concept, especially amongst dietitians in gastroenterology. Protein combining appears to remain a common misconception previously identified amongst RDs within the United States^23^ and other HCPs.^55^ The need for this to be addressed through continuing education is evident, as current research supports the sufficiency of plant proteins when a variety of sources are consumed and when energy needs are met.^56^, ^57^

Although the majority of RDs thought adopting a WFPB dietary pattern to be generally sustainable long‐term, most were unsure whether their clients with co‐morbidities would adhere to a WFPBD long‐term. Exploring this disparity further from open text‐box responses revealed certain RDs' reflections, indicating that it was based upon their individual clients' motivation (e.g., environment and health) and whether they had enough support in adopting a more PBD pattern as the main determinants. This highlights a potential area of focus for public health and behavioural change strategies when counselling patients choosing to adopt a more WFPB dietary pattern. RDs educated to postgraduate degree tended to exhibit higher knowledge scores, being more inclined to recommend WFPB diets, suggesting a correlation between further education and the likelihood of implementing WFPBDs.

### Limitations

Several limitations were identified in this study. It was apparent that gender discrepancies existed with participant demographics; being a higher proportion of female respondents (97%) and less representative of dietitians in this field (93%), particularly within the UK.^58^ Selection bias could not be completely ruled out amongst respondents ascribing to the MNW platform, given that it is an educational platform for HCPs that often focuses on sustainable nutrition, potentially generating more interest amongst RDs to engage in this topic. There appeared to be a higher percentage of RDs that identified as a flexitarian (30%) and vegan (7%) dietary pattern relative to the general population within the UK (approximately 13% and 4%)^59^ and ROI (19% and 2.6%),^60^ although statistics amongst RDs in the UK and ROI could differ from the general population. As this was a pooled analysis between the United Kingdom and Irish dietitians, we cannot completely disregard all potential differences between cohorts that were unable to be controlled for that may influence our findings (e.g., overall dietetic education and training, variations in healthcare systems, institutional policies, work environment and differences in patients' culture and adherence to dietary recommendations), which may impact differences in RDs' perspectives. Additionally, we acknowledge that RDs participating in this study may not be representative of RDs in the workforce as a whole, with fewer RD respondents specialising in certain common dietetic areas (e.g., renal, cardiothoracic, maternity, intensive care unit, nutrition support and sports nutrition). As the scoring system within the survey to assess RDs' cumulative knowledge of WFPB nutrition was not formally validated, the results should be interpreted with caution. In the regression analyses, personal dietary factors were not controlled for, given the lack of information on the personal motivation for respondents' dietary patterns (those following vegan or WFPBDs).

### Future recommendations and further research

Given the overall positive attitudes but significant barriers identified amongst this cohort of UK and Irish RDs in implementing WFPBDs, we propose several recommendations. The need to enhance access to further education and training for RDs on plant‐based nutrition is evident in order to further align with BDA's One Blue Dot and Eatwell recommendations for more SDPs. We suggest the integration of more comprehensive modules into dietetic curricula within universities while additionally offering continuing professional development courses for qualified RDs focusing on clinical guidance on PBD patterns, targeting specific clinical specialties. Recent evidence has already shown benefits in improving knowledge of plant‐based nutrition for other HCPs and positive health outcomes for their patients, with the integration of PBD curricula.^61^, ^62^ The development of more evidence‐based resources from public health agencies could further support RDs in counselling appropriate patients within a clinical framework, to help better navigate the transition to a more PBD pattern. These may include focusing on cooking skill workshops and demonstrations, with an emphasis on cost‐effectiveness within lower socioeconomic regions. Additionally, increasing institutional support for RDs and their patients, through key stakeholders in healthcare, may foster a more positive work environment that supports the integration of WFPBDs into patient care. We suggest future research into RDs' insights of perceived barriers at work when advocating for WFPBDs. While uncovering the beliefs related to WFPBDs in malnutrition risk, we recommend further research investigating differences in attitudes surrounding this amongst RDs specifically working within clinical practice, where concerns related to recommending PBDs for nutritionally at‐risk or more clinically complex patients appear to exist. Distinguishing this from RDs working in public health, who advocate for more general nutritional advice for the healthy population, may help identify key barriers amongst RDs with greater precision.

This cross‐sectional analysis provides unique insights into the perspectives of UK and Irish dietitians towards WFPBDs, highlighting overall positive attitudes yet considerable knowledge deficits and practical barriers to their potential broader implementation into clinical practice. Although the evidence and updated clinical guidance have become clear regarding the positive impact of PBDs on human and planetary health, it is apparent that there remains a significant need for further education, training and support within university settings, and amongst graduates and experienced RDs across clinical specialties.

## AUTHOR CONTRIBUTIONS

Michael Metoudi and Shireen Kassam contributed to the study design, data analysis, interpretation of the findings and the writing of the manuscript. Alexander Bauer contributed to the data analysis, interpretation of the findings and the writing of the manuscript. Tanya Haffner contributed to the study design, interpretation of the findings and the writing of the manuscript.

## CONFLICTS OF INTEREST STATEMENT

Michael Metoudi is a member of Plant‐Based Health Professionals United Kingdom. Shireen Kassam is the founder and director of Plant‐Based Health Professionals United Kingdom. Tanya Haffner is the founder of MyNutriWeb and Nutrilicious. The remaining author declares no conflict of interest.

### PEER REVIEW

The peer review history for this article is available at https://www.webofscience.com/api/gateway/wos/peer-review/10.1111/jhn.13386.

## ETHICS STATEMENT

This study protocol was reviewed and approved by the University of Winchester Ethics Review Board (HWB_REC_220405_Kassam).

## Supporting information

Supporting information.

## Data Availability

The data that support the findings of this study are available from the corresponding author upon reasonable request.
