# Supplementary material for: A cross‐sectional survey exploring knowledge, beliefs and barriers to whole food plant‐based diets amongst registered dietitians in the United Kingdom and Ireland
Source: J Hum Nutr Diet. 2024 Nov 3;38(1):e13386. doi: 10.1111/jhn.13386 (PMC11589407; doi:10.1111/jhn.13386)
Supplement: Supplementary file 1 — Supporting information. [file JHN-38-0-s001.docx]

**Supplementary Material**

**Section 1 - Additional figures**

**
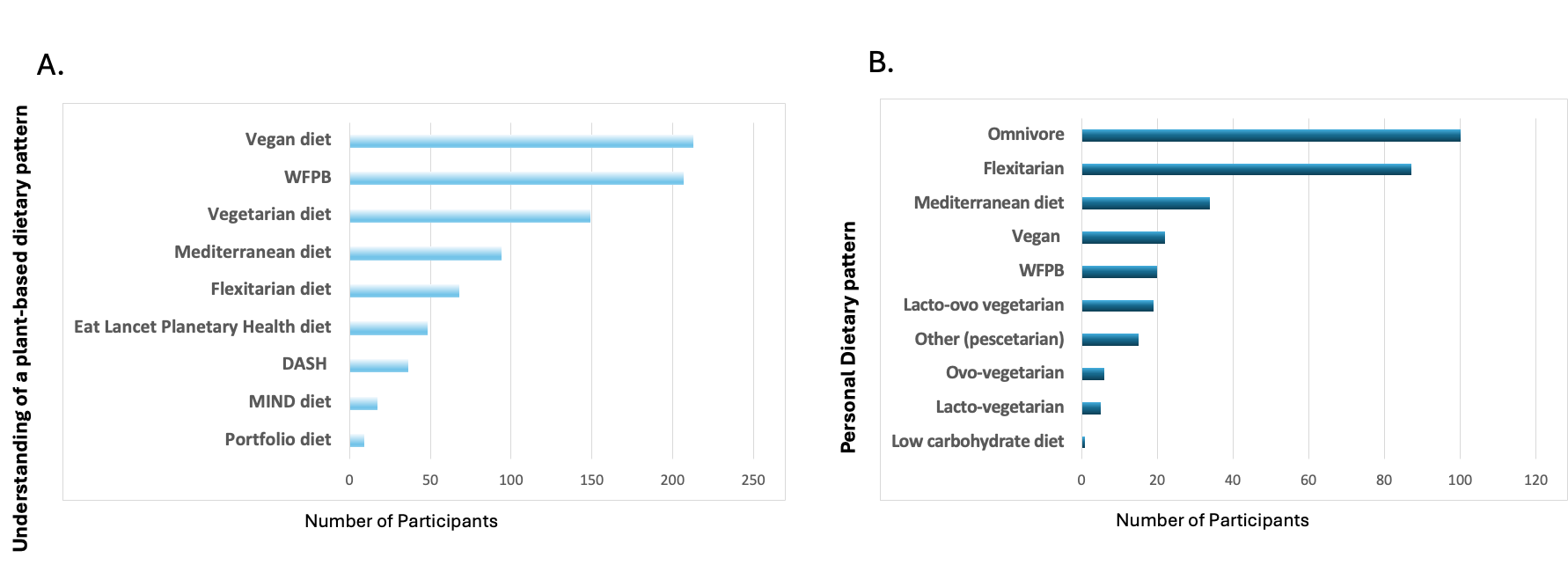
**

**
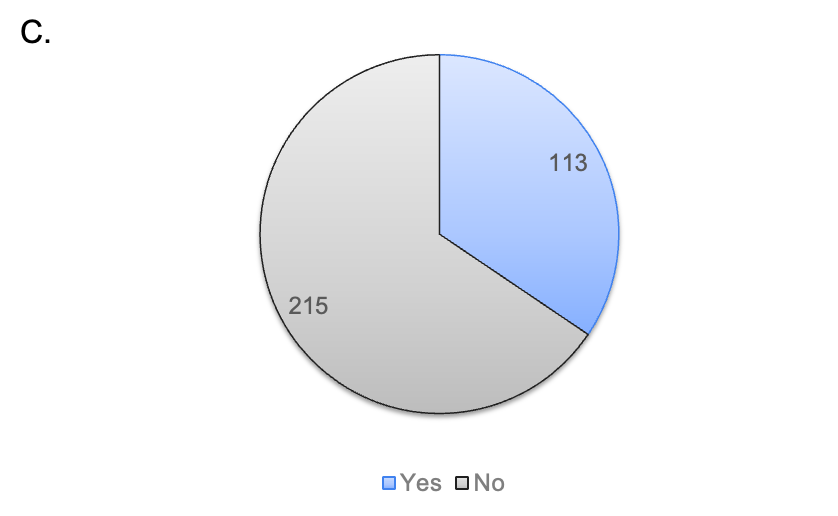
**

**Figure S1. Understanding of a WFPB diet amongst dietitians.** (A) Showing the commonest response from RDs on their understanding of what constitutes a plant-based dietary pattern. (B) Personal dietary pattern amongst RD participants (n = 335). (C) Number of respondents personally tried to transition to a WFPBD.

**Figure S2 Devised Scoring system assessing knowledge of WFPBDs amongst RDs**

**
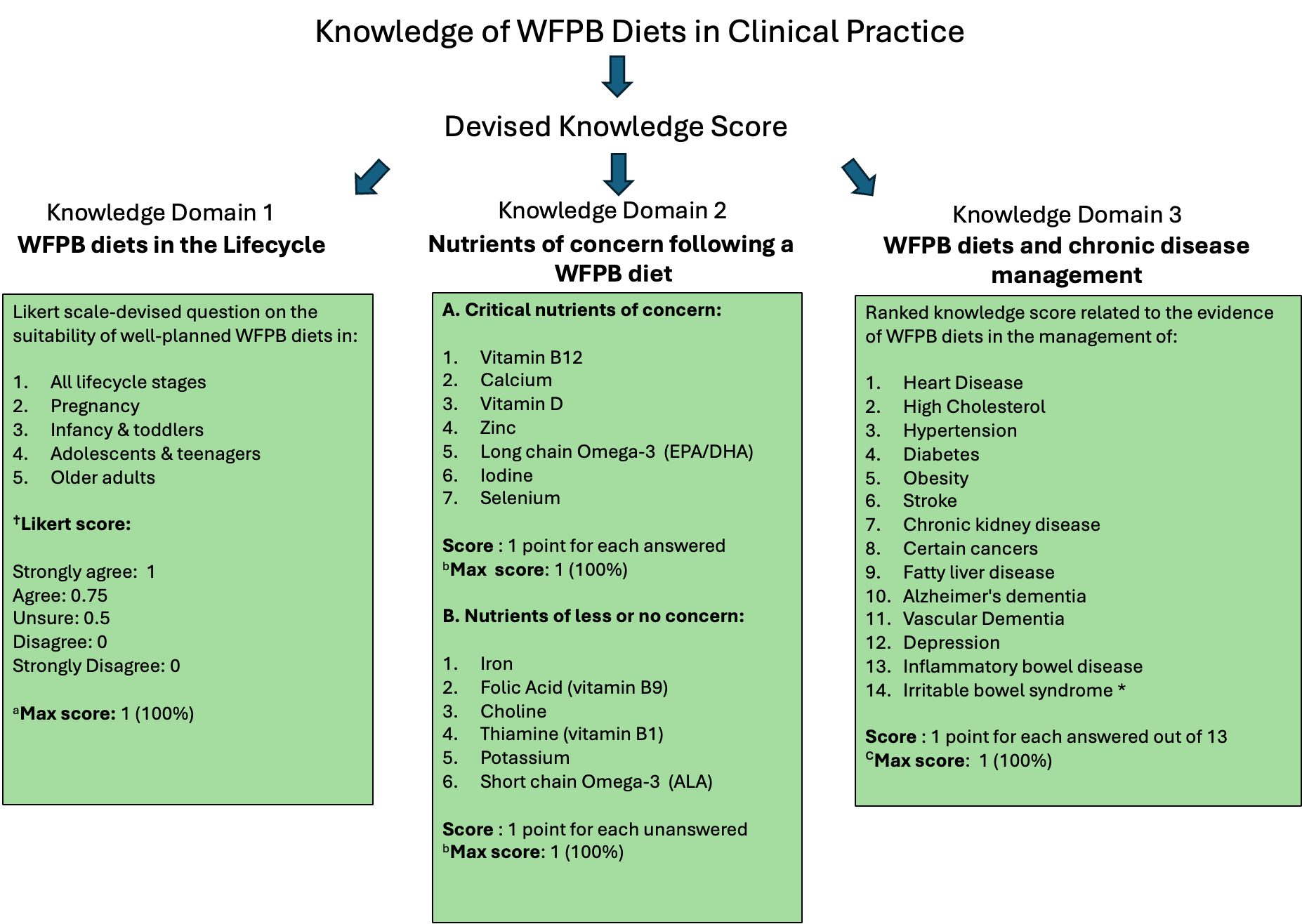
**

**Figure S2 knowledge assessment of WFPBDs in clinical practice.** Participant’s knowledge was assessed based on 3 central knowledge domains related to: i) the suitability of a well-planned WFPBD throughout each of the lifecycle stages, ii) nutrients of concern following a WFPBD and iii) current clinical evidence of WFPBDs in the management of chronic lifecycle related diseases. ^a,b,c^Knowledge scores were calculated by taking an average of the total participants’ response and normalising to a scale for equal weighting between the knowledge domains (with each score lying between 0 and 1, 1 being the max score). ♱Likert scores with the lowest response category ‘No, strongly disagree’ encoded as 0, and the highest ‘Yes, strongly agree’ encoded as 1) and dividing the resulting average by 5. Scores were controlled for age, education, years of practice and education in WFPB nutrition.

**Supplementary Figure S3 Current clinical evidence of WFPBDs in chronic disease**

**management**

**
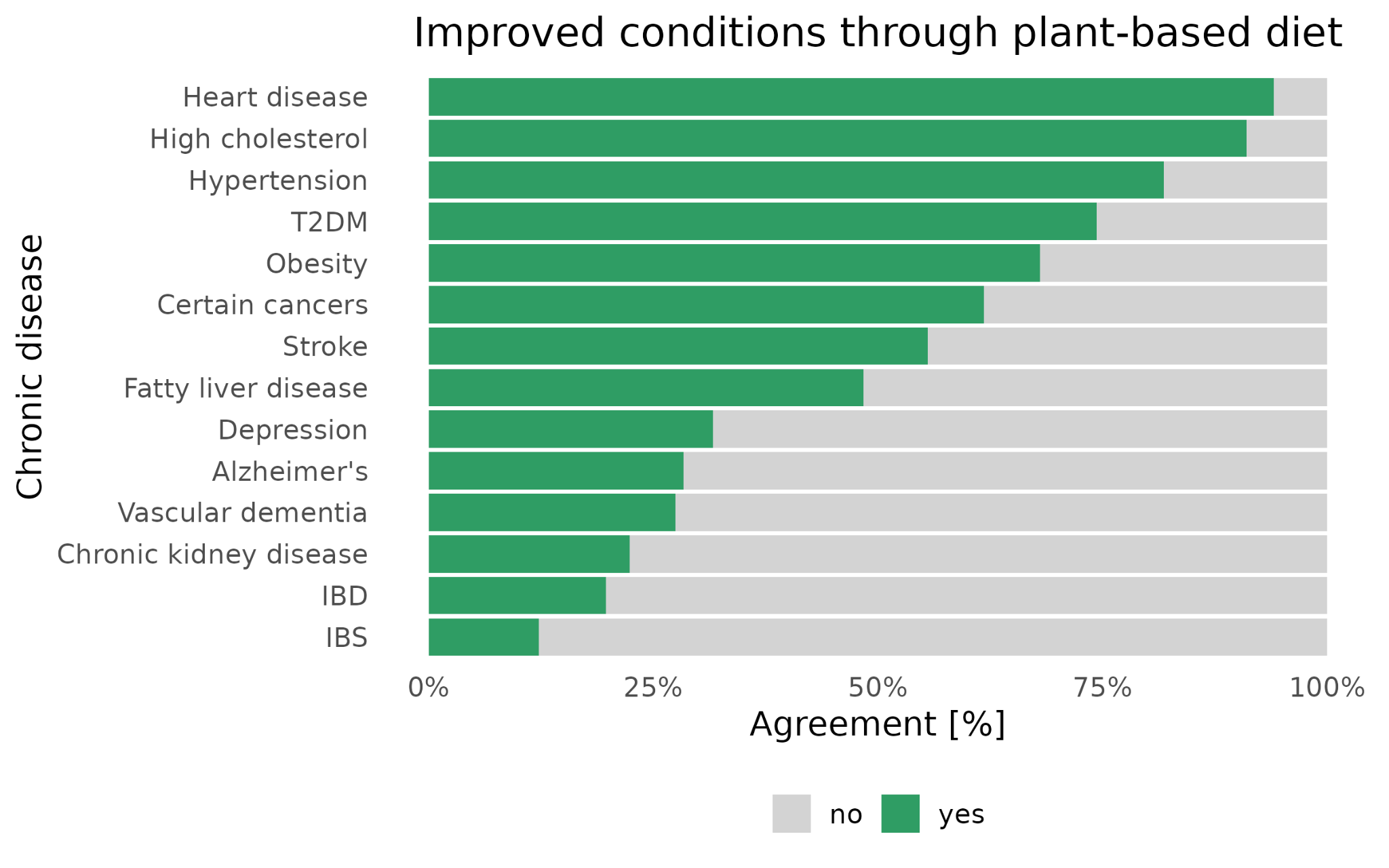
**

**Figure S3. Knowledge of WFPBDs in chronic disease risk.** Highlighting the commonest response amongst RD participants’ knowledge on the role of WFPBDs in reducing the risk and/or management of the leading chronic diseases.

**Supplementary Figure S4 Nutrients of concern on a WFPBD**

**
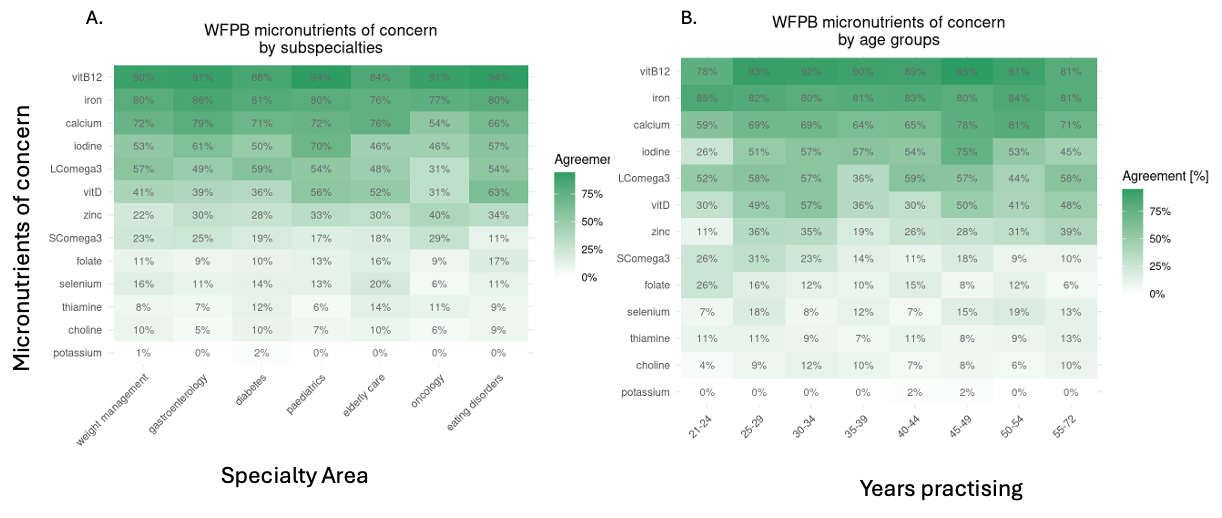
**

**Figure S4. Heat map showing the main micronutrients of concern on a WFPBD amongst RDs based on (A) area of specialty and (B) years of practice.**

**Supplementary Figure S5 Recommending a WFPBD in clinical practice**

**
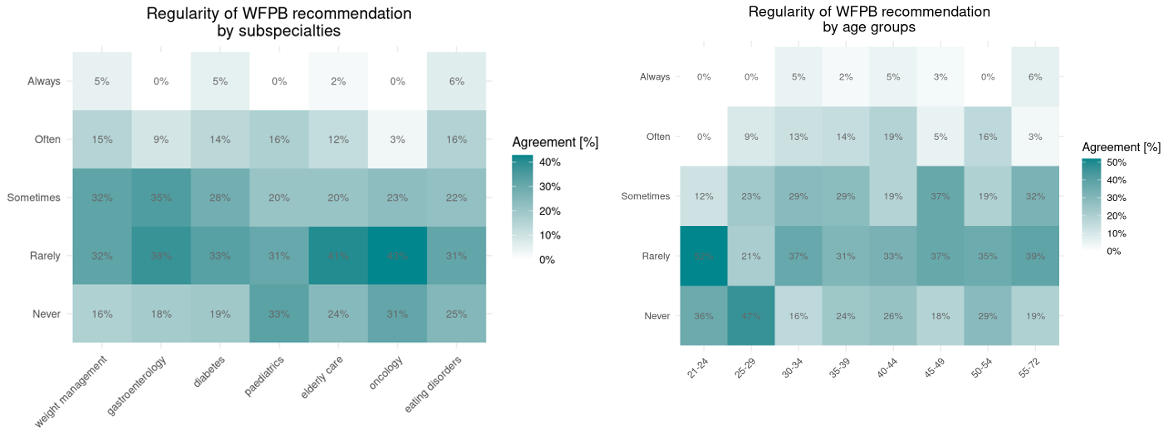
**

**Figure S5. Heat map showing the frequency of recommending a WFPBD amongst RDs; based on area of specialty and years of practice.**

**Supplementary Figure S6 Perceived patient’s motivations for switching to a WFPBD**

**
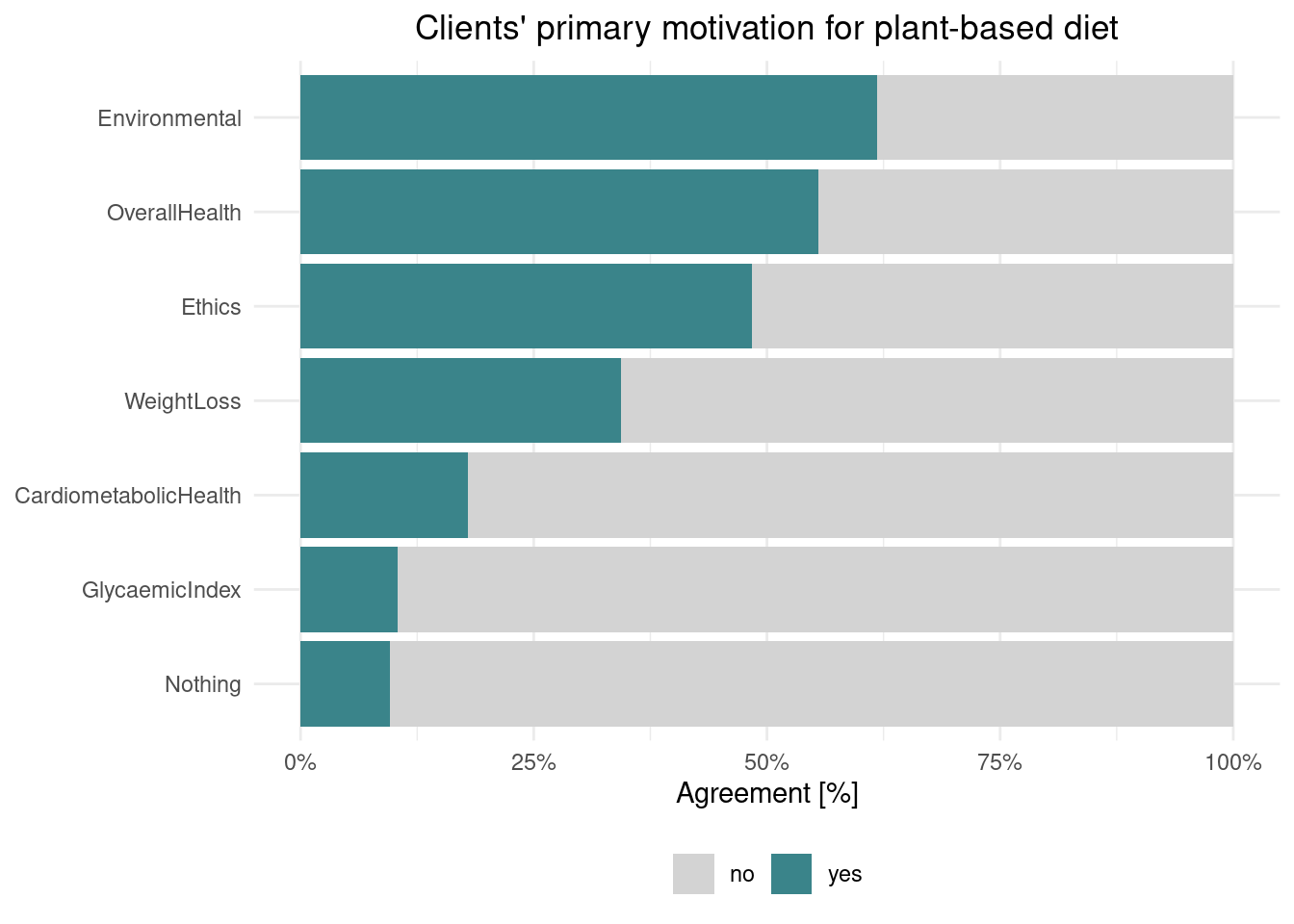
**

**Figure S6. Client’s perceived motivations for switching to a WFPBD.** Highlighting the commonest response amongst RD participants related to perceived patient’s motivations in switching to a WFPBD.

**Supplementary Figure S7 RD’s beliefs of WFPBDs in individuals of lower Socioeconomic status**

**
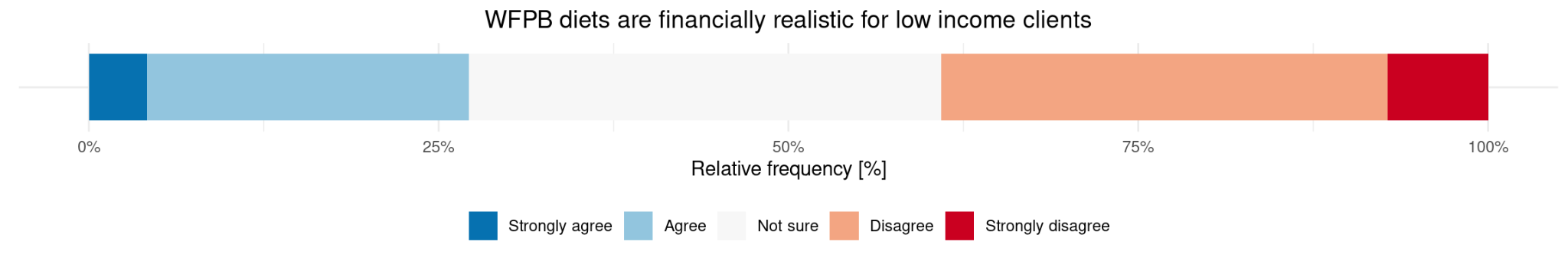
**

**Fig S7. Likert scale assessment on RD’s beliefs on whether they think WFPBDs are a viable dietary pattern in individuals of lower Socioeconomic status (SES).**

**Supplementary Fig S8 RD’s beliefs regarding the likelihood of their clients with comorbidities adhering to a WFPBD**

**
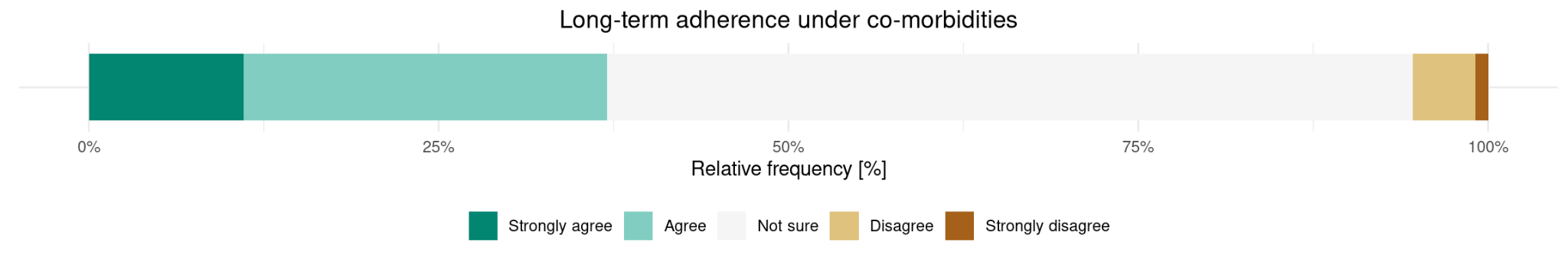
**

**Fig S8. Likert scale assessment of RD’s beliefs regarding the likelihood of their clients with comorbidities adhering to a WFPBD.**

**Supplementary Figure S9 Thematic analyses on RD’s beliefs of WFPB diets in clinical practice**

**
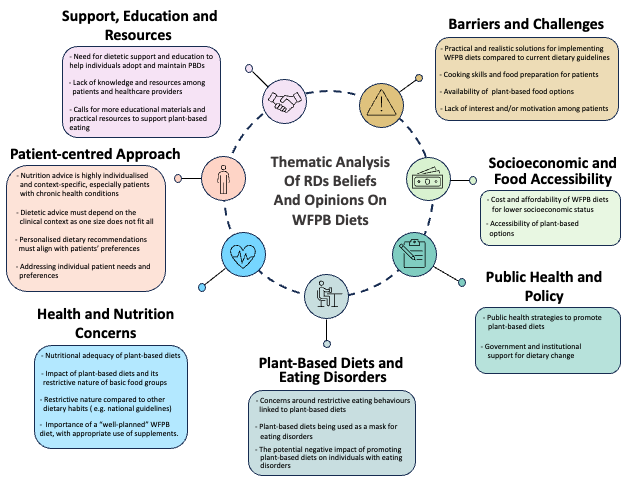
**

**Figure S9. Qualitative thematic analysis highlighting 7 core themes related to RDs’ beliefs and attitudes towards WFPBDs from an open-text box response.**

**Section 2 - Logistic and Linear Regression analysis tables**

**Table S1. Linear regression model estimates on the Knowledge Domain 1 score (stages of the life cycle). Estimates are shown together with 95% confidence intervals (95% CI).**

| **Parameter** | **Estimate** | **95% CI** | **^a^p-value** |
| --- | --- | --- | --- |
| **^b^Intercept** | 0.65 | [0.57, 0.72] |  |
| **Area of specialty** |  |  |  |
| **Weight management** | +0.05 | [-0.02, +0.12] | 0.1366 |
| **Gastroenterology** | -0.03 | [-0.10, +0.04] | 0.403 |
| **Diabetes** | -0.004 | [-0.08, +0.08] | 0.9915 |
| **Paediatrics** | +0.0032 | [-0.07, +0.08] | 0.9326 |
| **Care for the elderly** | -0.006 | [-0.08, +0.07] | 0.8772 |
| **Oncology** | -0.0889 | [-0.18, -0.003] | 0.05 * |
| **Eating disorders** | +0.06 | [-0.03, +0.15] | 0.2064 |
| **^c^Years of practice** | -0.017 | [-0.05, +0.01] | 0.2277 |
| **Education** |  |  |  |
| **Postgraduate degree** | +0.0037 | [-0.05, +0.06] | 0.8984 |
| **PhD** | -0.07 | [-0.2, +0.06] | 0.3104 |
| **Education in WFPB nutrition** | -0.009 | [-0.04, +0.02] | 0.4763 |

^a^Significant effects (p < 0.05) are marked ‘*’.

^b^The intercept reflects the average score for a dietitian with (i) an area of specialty outside of the above listed ones, (ii) zero years of practice, and (iii) an undergraduate degree.

^c^Effect estimates relate to an increase of the years of practice by 10 years.

**Table S2. Linear regression model estimates on the Knowledge Domain 2a score (micronutrients of concern). Estimates are shown together with 95% confidence intervals (95% CI).**

| **Parameter** | **Estimate** | **95% CI** | **^a^p-value** |
| --- | --- | --- | --- |
| **^b^Intercept** | 0.49 | [0.42, 0.56] |  |
| **Area of specialty** |  |  |  |
| **Weight management** | +0.0046 | [-0.06, +0.06] | 0.8811 |
| **Gastroenterology** | +0.017 | [-0.05, +0.08] | 0.6167 |
| **Diabetes** | -0.080 | [-0.08, +0.06] | 0.8206 |
| **Paediatrics** | +0.07 | [+0.003, +0.14] | 0.0411 * |
| **Care for the elderly** | +0.018 | [-0.05, +0.09] | 0.6083 |
| **Oncology** | -0.07 | [-0.15, +0.06] | 0.0704 |
| **Eating disorders** | +0.05 | [-0.04, +0.13] | 0.2589 |
| **^c^Years of practice** | +0.001 | [-0.02, +0.03] | 0.937 |
| **Education** |  |  |  |
| **Postgraduate degree** | +0.009 | [-0.04, +0.06] | 0.7304 |
| **PhD** | +0.03 | [-0.09, +0.15] | 0.5058 |
| **Education in WFPB nutrition** | -0.007 | [-0.03, +0.02] | 0.5289 |

^a^Significant effects (p < 0.05) are marked ‘*’.

^b^The intercept reflects the average score for a dietitian with (i) an area of specialty outside of the above listed ones, (ii) zero years of practice, and (iii) an undergraduate degree.

^c^Effect estimates relate to an increase of the years of practice by 10 years.

**Table S3. Linear regression model estimates on the Knowledge Domain 2b score (non-critical micronutrients). Estimates are shown together with 95% confidence intervals (95% CI).**

| **Parameter** | **Estimate** | **95% CI** | **^a^p-value** |
| --- | --- | --- | --- |
| **^b^Intercept** | 0.74 | [0.70, 0.78] |  |
| **Area of specialty** |  |  |  |
| **Weight management** | +0.001 | [-0.04, +0.04] | 0.948 |
| **Gastroenterology** | +0.002 | [-0.04, +0.04] | 0.9396 |
| **Diabetes** | -0.01 | [-0.05, +0.03] | 0.6788 |
| **Paediatrics** | +0.01 | [-0.03, +0.06] | 0.4845 |
| **Care for the elderly** | +0.006 | [-0.04, +0.05] | 0.7742 |
| **Oncology** | +0.002 | [-0.05, +0.05] | 0.9483 |
| **^c^Eating disorders** | +0.08 | [-0.04, +0.05] | 0.7305 |
| **Years of practice** | +0.02 | [+0.0005, +0.03] | 0.0441 * |
| **Education** |  |  |  |
| **Postgraduate degree** | +0.03 | [+0.002, +0.06] | 0.0409* |
| **PhD** | +0.04 | [-0.03, +0.11] | 0.2845 |
| **Education in WFPB nutrition** | -0.002 | [-0.01, +0.01] | 0.9738 |

^a^Significant effects (p < 0.05) are marked ‘*’.

^b^The intercept reflects the average score for a dietitian with (i) an area of specialty outside of the above listed ones, (ii) zero years of practice, and (iii) an undergraduate degree.

^c^Effect estimates relate to an increase of the years of practice by 10 years.

**Table S4 Linear regression model estimates on the Knowledge Domain 3 score (chronic disease management). Estimates are shown together with 95% confidence intervals (95% CI).**

| **Parameter** | **Estimate** | **95% CI** | **^a^p-value** |
| --- | --- | --- | --- |
| **^b^Intercept** | 0.56 | [0.48, 0.63] |  |
| **Area of specialty** |  |  |  |
| **Weight management** | +0.04 | [-0.03, +0.10] | 0.2699 |
| **Gastroenterology** | +0.04 | [-0.03, +0.11] | 0.2448 |
| **Diabetes** | +0.02 | [-0.05, +0.10] | 0.52 |
| **Paediatrics** | +0.06 | [-0.007, +0.14] | 0.0719 |
| **Care for the elderly** | -0.008 | [-0.08, +0.07] | 0.8286 |
| **Oncology** | -0.04 | [-0.13, +0.05] | 0.3632 |
| **Eating disorders** | +0.07 | [-0.02, +0.16] | 0.1243 |
| **^c^Years of practice** | -0.016 | [-0.04, +0.01] | 0.2456 |
| **Education** |  |  |  |
| **Postgraduate degree** | +0.04 | [-0.01, +0.10] | 0.1175 |
| **PhD** | -0.003 | [-0.13, +0.12] | 0.9655 |
| **Education in WFPB nutrition** | -0.03 | [-0.06, -0.006] | 0.0165* |

^a^Significant effects (p < 0.05) are marked ‘*’.

^b^The intercept reflects the average score for a dietitian with (i) an area of specialty outside of the above listed ones, (ii) zero years of practice, and (iii) an undergraduate degree.

^c^Effect estimates relate to an increase of the years of practice by 10 years.

**Table S5 Logistic regression model estimates on the evaluation of RDs knowledge on plant protein sufficiency (complete essential amino acids), modelling the binarised Likert scale ‘Yes, (strongly) agree’ vs. ‘Not sure / No, (strongly) disagree’. Positive effects encode a higher chance of agreement. Odds ratio estimates are shown together with 95% confidence intervals (95% CI).**

| **Parameter** | **Estimate** | **95% CI** | **^a^p-value** |
| --- | --- | --- | --- |
| **^b^Intercept** | 2.37 | [1.16, 4.82] |  |
| **Area of specialty** |  |  |  |
| **Weight management** | -33% | [-64%, +22%] | 0.1862 |
| **Gastroenterology** | +151% | [+10%, +464%] | 0.0282 * |
| **Diabetes** | -12% | [-56%, +76%] | 0.7122 |
| **Paediatrics** | +44% | [-31%, +201%] | 0.3342 |
| **Care for the elderly** | -3% | [-54%, +103%] | 0.9306 |
| **Oncology** | -57% | [-80%, -8%] | 0.0305* |
| **Eating disorders** | +44% | [-445, +271] | 0.448 |
| **^c^Years of practice** | -10% | [-30%, +17%] | 0.4499 |
| **Education** |  |  |  |
| **Postgraduate degree** | -14% | [-50%, +47%] | 0.5795 |
| **PhD** | -47% | [-83%, +65%] | 0.2716 |
| **Education in WFPB nutrition** | 33% | [+4%, +71%] | 0.022* |

^a^Significant effects (p < 0.05) are marked ‘*’.

^b^The intercept reflects the average odds (for agreement vs. non-agreement) for a dietitian with (i) an area of specialty outside of the above listed ones, (ii) zero years of practice, and (iii) an undergraduate degree.

^c^Effect estimates relate to an increase of the years of practice by 10 years.

AUC: 0.64

**Table S6 Logistic regression model estimates on the evaluation of the binary question if RDs would recommend or would not recommend a WFPBD for type-2 diabetes mellitus (T2DM) patients. Positive effects encode a higher chance of recommendation. Odds ratio estimates are shown together with 95% confidence intervals (95% CI).**

| **Parameter** | **Estimate** | **95% CI** | **^a^p-value** |
| --- | --- | --- | --- |
| **^b^Intercept** | 0.22 | [0.10, 0.48] |  |
| **Area of specialty** |  |  |  |
| **Weight management** | +70% | [-9%, +217%] | 0.0993 |
| **Gastroenterology** | +7% | [-47%, +119%] | 0.8433 |
| **Diabetes** | -14% | [-60%, +84%] | 0.6929 |
| **Paediatrics** | +15% | [-43%, +135%] | 0.7007 |
| **Care for the elderly** | +24% | [-41%, +160%] | 0.5704 |
| **Oncology** | -41% | [-78%, +62%] | 0.3509 |
| **Eating disorders** | +145% | [+12%, +434%] | 0.0238* |
| **^c^Years of practice** | -10% | [-32%, +20%] | 0.4663 |
| **Education** |  |  |  |
| **Postgraduate degree** | +84% | [+4%, +227%] | 0.0363 * |
| **PhD** | +95% | [-44%, +582%] | 0.297 |
| **Education in WFPB nutrition** | -25% | [-24%, +10%] | 0.2185 |

^a^Significant effects (p < 0.05) are marked ‘*’.

^b^The intercept reflects the average odds (for recommendation vs. no recommendation) for a dietitian with (i) an area of specialty outside of the above listed ones, (ii) zero years of practice, and (iii) an undergraduate degree.

^c^Effect estimates relate to an increase of the years of practice by 10 years.

AUC: 0.61

**Table S7 Logistic regression model estimates on the evaluation of the binary question if RDs would recommend or would not recommend a WFPBD for cardiovascular disease(CVD) patients. Positive effects encode a higher chance of recommendation. Odds ratio(OR) estimates are shown together with 95% confidence intervals (95% CI).**

| **Parameter** | **Estimate** | **95% CI** | **^a^p-value** |
| --- | --- | --- | --- |
| **^b^Intercept** | 0.30 | [0.14, 0.64] |  |
| **Area of specialty** |  |  |  |
| **Weight management** | +183% | [+54%, +420%] | <0.0001* |
| **Gastroenterology** | -2% | [-53%, +107%] | 0.964 |
| **Diabetes** | -0.4% | [-51%, +105%] | 0.9919 |
| **Paediatrics** | -39% | [-72%, +36%] | 0.2259 |
| **Care for the elderly** | -54% | [-81%, +12%] | 0.0872 |
| **Oncology** | -59% | [-86%, +24%] | 0.1142 |
| **Eating disorders** | +96% | [-13%, +346%] | 0.1062 |
| **^c^Years of practice** | -10% | [-33%, +19%] | 0.445 |
| **Education** |  |  |  |
| **Postgraduate degree** | +152% | [+40%, +353%] | 0.002 * |
| **PhD** | +21% | [-70%, +402%] | 0.7876 |
| **Education in WFPB nutrition** | -27% | [-43%, -5%] | 0.0168* |

^a^Significant effects (p < 0.05) are marked ‘*’.

^b^The intercept reflects the average odds (for recommendation vs. no recommendation) for a dietitian with (i) an area of specialty outside of the above listed ones, (ii) zero years of practice, and (iii) an undergraduate degree.

^c^Effect estimates relate to an increase of the years of practice by 10 years.

AUC: 0.68

**Table S8 Logistic regression model estimates on the evaluation of the binary question if RDs would recommend or would not recommend a WFPBD for weight loss. Positive effects encode a higher chance of recommendation. OR estimates are shown together with 95% confidence intervals (95% CI).**

| **Parameter** | **Estimate** | **95% CI** | **ap-value** |
| --- | --- | --- | --- |
| **^b^Intercept** | 0.35 | [0.16, 0.75] |  |
| **Area of specialty** |  |  |  |
| **Weight management** | +137% | [+14%, +299%] | 0.0172 * |
| **Gastroenterology** | +5% | [-50%, +120%] | 0.8939 |
| **Diabetes** | -8% | [-48%, +126%] | 0.8433 |
| **Paediatrics** | -6% | [-54%, +125%] | 0.8781 |
| **Care for the elderly** | -15% | [-61%, +87%] | 0.6858 |
| **Oncology** | -62% | [-91%, +10%] | 0.07 |
| **Eating disorders** | +239% | [+54%, +645%] | 0.0024* |
| **cYears of practice** | -20% | [-40%, +7%] | 0.1347 |
| **Education** |  |  |  |
| **Postgraduate degree** | +81% | [+2%, +221%] | 0.0418 * |
| **PhD** | -33% | [-84%, +279%] | 0.7454 |
| **Education in WFPB nutrition** | -29% | [-45%, -8%] | 0.01* |

^a^Significant effects (p < 0.05) are marked ‘*’.

^b^The intercept reflects the average odds (for recommendation vs. no recommendation) for a dietitian with (i) an area of specialty outside of the above listed ones, (ii) zero years of practice, and (iii) an undergraduate degree.

^c^Effect estimates relate to an increase of the years of practice by 10 years.

AUC: 0.62

**Table S9 Logistic regression model estimates on the evaluation of RDs belief on the long-term sustainability and adherence of WFPBDs, modelling the binarised Likert scale ‘(Strongly) Agree’ vs. ‘Not sure / (Strongly) Disagree’. Positive effects encode a higher chance of agreement. Odds ratio estimates are shown together with 95% confidence intervals (95% CI).**

| **Parameter** | **Estimate** | **95% CI** | **^a^p-value** |
| --- | --- | --- | --- |
| **^b^Intercept** | 1.59 | [0.86,3.31] |  |
| **Area of specialty** |  |  |  |
| **Weight management** | +3% | [-43%, +87%] | 0.913 |
| **Gastroenterology** | +70% | [-14%, +234%] | 0.1265 |
| **Diabetes** | +11% | [-44%, +120%] | 0.7641 |
| **Paediatrics** | +24% | [-36%, +140%] | 0.5258 |
| **Care for the elderly** | +26% | [-37%, +153%] | 0.5049 |
| **Oncology** | -63% | [-81%, -19%] | 0.00118 * |
| **Eating disorders** | +2% | [+53%, +125%] | 0.9438 |
| **^c^Years of practice** | -3% | [-24%, +24%] | 0.7973 |
| **Education** |  |  |  |
| **Postgraduate degree** | +73% | [+5%, +183%] | 0.0302 * |
| **PhD** | +52% | [-52%, +375%] | 0.4734 |
| **Education in WFPB nutrition** | -7% | [-25%, +17%] | 0.5472 |

^a^Significant effects (p < 0.05) are marked ‘*’.

^b^The intercept reflects the average odds (for agreement vs. non-agreement) for a dietitian with (i) an area of specialty outside of the above listed ones, (ii) zero years of practice, and (iii) an undergraduate degree.

^c^Effect estimates relate to an increase of the years of practice by 10 years.

AUC: 0.59

**Table S10 Logistic regression model estimates on the evaluation of RDs attitude towards recommending WFPBDs in clinical practice, modelling the binarised Likert scale ‘Always / Often / Sometimes’ vs. ‘Rarely / Never’. Positive effects encode a higher chance of recommendation. Odds ratio estimates are shown together with 95% confidence intervals (95% CI).**

| **Parameter** | **Estimate** | **95% CI** | **^a^p-value** |
| --- | --- | --- | --- |
| **^b^Intercept** | 0.12 | [0.05, 0.29] |  |
| **Area of specialty** |  |  |  |
| **Weight management** | +84% | [+2%, +230%] | 0.0426 * |
| **Gastroenterology** | +32% | [-30%, +153%] | 0.3977 |
| **Diabetes** | +17% | [-40%, +128%] | 0.6539 |
| **Paediatrics** | -20% | [-59%, +56%] | 0.5142 |
| **Care for the elderly** | -1% | [-51%, +98%] | 0.9702 |
| **Oncology** | -39% | [-78%, +19%] | 0.1205 |
| **Eating disorders** | +23% | [-44%, +171%] | 0.6126 |
| **^c^Years of practice** | +25% | [-3%, +60%] | 0.0757 |
| **Education** |  |  |  |
| **Postgraduate degree** | +145% | [+47%, +309%] | 0.0004 * |
| **PhD** | +145% | [+47%, +309%] | 0.0109 * |
| **Barriers** |  |  |  |
| **WFPB nutrition education in university** | -7% | [-25%, +18%] | 0.5594 |
| **Availability WFPB nutrition education resources** | +11% | [-15%, +47%] | 0.4147 |
| **Workplace support if recommending WFPBDs** | +39% | [+8%, +79%] | 0.0093 * |

^a^Significant effects (p < 0.05) are marked ‘*’.

^b^The intercept reflects the average odds (for agreement vs. non-agreement) for a dietitian with (i) an area of specialty outside of the above listed ones, (ii) zero years of practice, and (iii) an undergraduate degree.

^c^Effect estimates relate to an increase of the years of practice by 10 years.

AUC: 0.61

**Section 3 - Thematic analysis to open-textbox responses**

**Table S11 Demographics of respondents who answered to the open-ended question analysed in supplementary table S12.**

| **Demographic** | **Total (%)** |
| --- | --- |
| **Median age (years)** | 38 |
| **Education** |  |
| **Bachelor's degree** | 36(41%) |
| **Post-graduate** | 44(52%) |
| **PhD** | 6(7%) |
| **Area of work** |  |
| **Hospital** | 27(31%) |
| **Primary care/ Community** | 46(53%) |
| **Private Practice** | 15(17%) |
| **Academia/**  **Research** | 13(15%) |
| **Public Health** | 7(8%) |
| **Area of specialty** |  |
| **Weight management** | 16(19%) |
| **Diabetes** | 17(20%) |
| **Gastroenterology** | 16(19%) |
| **Paediatrics** | 7(8%) |
| **Care for the elderly** | 15(17%) |
| **Oncology** | 7(8%) |
| **Dietary pattern** |  |
| **Omnivorous** | 20 (23%) |
| **Mediterranean** | 10 (12%) |
| **Flexitarian** | 33(38%) |
| **Vegetarian** | 9(10%) |
| **WFPB** | 9(10%) |
| **Vegan** | 5(6%) |

**Table S12 Analysis of the open-ended question: Selected keywords and number of matches from all extracted comments**

| **^a^Key words** | **^b^Frequency** |
| --- | --- |
| **Plant-based diet** | **34** |
| **Patient(s)** | **29** |
| **Support** | **15** |
| **Cooking** | **11** |
| **Knowledge** | **9** |
| **Resources** | **9** |
| **Eating Disorder(s)** | **8** |
| **Education** | **8** |
| **Socioeconomic** | **7** |
| **Barrier(s)** | **6** |
| **Patient-centred** | **4** |
| **Restrictive** | **3** |

Total comments analysed: 87

^a^Keyword was extracted once per total individual respondent’s comment if there were

multiples of the same key word in each respondent’s comment.

^b^Total number of times words appeared within the open text-box responses.

**Table S13 Inductive codes and themes from the open-ended question**

| **Code** | **Themes** | **^a^Frequency of recurring theme (%)** |
| --- | --- | --- |
| **Barriers and challenges to practical implementation** | **Practical solutions for implementing and adopting plant-based diets**  **Realistic approaches to dietary changes compared to standard national dietary guidelines or other diets**  **Too extreme compared to other diets**  **Cooking skills and food preparation for patients.**  **Availability of plant-based options**  **Lack of interest and/or motivation among clients** | **35(40%)** |
| **Support, education and resources** | **Need for dietetic support and education to help individuals adopt and maintain plant-based diets.**  **Lack of knowledge and resources among patients and healthcare providers on plant-based nutrition.**  **Calls for more educational materials and practical resources to support plant-based eating.** | **21 (24%)** |
| **Patient-centred approach** | **Nutrition advice is highly individualised and context-specific, especially patients with chronic health conditions.**  **Must depend on the clinical context**  **Personalised dietary recommendations must align with patients’ preferences**  **Addressing individual patient needs and preferences** | **13 (15%)** |
| **Health and nutrition concerns** | **Nutritional adequacy of plant-based diets**  **Impact of plant-based diets on restrictive nature of basic food groups**  **Restrictive nature compared to other dietary habits** | **13 (15%)** |
| **Plant-based diets and eating disorders** | **Concerns about restrictive eating behaviours linked to plant-based diets.**  **Plant-based diets being used as a mask for eating disorders.**  **The potential negative impact of promoting plant-based diets on individuals with eating disorders.** | **10 (11%)** |
| **Socioeconomic and food Accessibility Issues** | **Cost and affordability of plant-based diets**  **Accessibility of plant-based options in different settings** | **10(11%)** |
| **Public health and policy** | **Public health strategies to promote plant-based diets**  **Government and institutional support for dietary change** | **6 (7%)** |

Total usable comments: 86

^a^Frequency of themes were calculated by the number of times the themes occurred in the total count of responses

**Section 4 - Dietitian Survey**

**Dietitian survey on evaluating RDs perspective; knowledge, beliefs and barriers on WFPBDs among RDs within the UK and ROI.**

**Consent**

**1.Do you consent to take part in this survey?.**

**Yes**

**No**

**Section 1 Participant Details**

**Please fill in the following demographic information**

**2.Are you a registered dietitian?**

**Yes**

**No**

**3.What is your age (in years)? Required to answer.**

**4.Which of the following best describes you? (Please specify)**

**Male**

**Female**

**Non-binary**

**Prefer not to say**

**Other**

**5.What is your current education level?**

**Undergraduate degree**

**Post-graduate diploma**

**Master’s degree**

**PhD**

**Other**

**6. How long have you been working as a dietitian? (Please specify in years)**

**7. What country are you currently working in as a dietitian?**

**8. What area of dietetics do you work in? (choose multiple if applicable)**

**Academia**

**Catering**

**Community**

**Hospital**

**Industry**

**Primary care**

**Private practice**

**Public health**

**Research**

**Other**

**9.What is your subspecialty? (choose multiple if applicable)**

**Cardiothoracic**

**Care for the elderly**

**Diabetes**

**Gastroenterology**

**Intensive care unit**

**Long-term care facility**

**Maternity**

**Obesity**

**Oncology**

**Paediatrics**

**Psychiatry/eating disorders**

**Renal**

**Sports nutrition**

**Transplantation**

**Weight management**

**Other**

**10.Which of the following do you consider best describes a plant-based dietary pattern? (choose multiple options if applicable)**

**DASH diet (Dietary Approaches to Stop Hypertension)**

**Eat Lancet Planetary Health diet**

**Mediterranean diet**

**Flexitarian diet**

**MIND diet (Mediterranean-DASH Intervention for Neurodegenerative Delay)**

**Portfolio diet**

**Vegan diet**

**Vegetarian diet**

**Whole food plant-based diet (Meat is limited or excluded completely and whole plant foods are encouraged)**

**Other**

**11.How would you describe your dietary pattern?**

**Omnivore**

**Flexitarian**

**Lacto-ovo vegetarian**

**Lacto-vegetarian**

**Ovo-vegetarian**

**Whole food plant-based (WFPB) diet**

**Mediterranean diet**

**Vegan**

**Low carbohydrate diet**

**Other**

**Section 2 - This section aims to explore your current knowledge of plant-based nutrition.**

**The rest of the survey refers to a predominantly whole food plant-based (WFPB) diet, defined within the framework of the Eat Lancet Planetary Health Diet, which is dominated (>85% of energy from minimally processed whole plant foods e.g. fruits, vegetables, legumes, whole grains, nuts, seeds, herbs and spices) and where meat and dairy foods are minimised.**

**12. The following questions relate to the current knowledge of whole food plant-based diets throughout the stages of the life cycle(5-point Likert scale, strongly agree, agree, not sure, disagree, strongly disagree).**

**i) A well-planned whole food plant-based diet is suitable and healthy for all life stages.**

**ii) A well-planned whole food plant-based diet is suitable in all stages of pregnancy and lactation.**

**iii) It is possible for children (infants and toddlers) to meet all nutritional requirements on a well-planned whole food plant-based diet.**

**iv) It is possible for adolescents and teenagers to meet all nutritional requirements on a well-planned whole food plant-based diet**

**v) It is difficult for older persons to achieve their energy and protein requirements on a well-planned, whole food plant-based diet?**

**vi) Plant proteins are considered to be an incomplete source of protein (i.e. they do not contain all the essential amino acids) and as such should be carefully paired with other sources of plant protein**

**13.Which of the following would you consider to be micronutrients of concern on a whole food plant-based diet (i.e. associated with an increased risk of deficiency) (please choose multiple answers if applicable)?**

**Calcium**

**Choline**

**Folate**

**Iodine**

**Iron**

**Long-chain omega-3 fatty acids (EPA/DHA)**

**Potassium**

**Selenium**

**Short chain omega-3 fatty acids (ALA)**

**Vitamin B12**

**Vitamin D**

**Thiamine**

**Zinc**

**Other**

**14.Which of the following conditions has a plant-based diet been shown to reduce the risk or improve the management of? (Choose multiple answers if applicable).**

**Heart disease**

**High cholesterol**

**Hypertension**

**Type 2 Diabetes Mellitus**

**Obesity**

**Stroke**

**Chronic kidney disease**

**Certain cancers**

**Inflammatory bowel disease**

**Irritable bowel syndrome**

**Fatty liver disease**

**Alzheimer dementia**

**Vascular dementia**

**Depression**

**Other**

**Section 3 - Beliefs And Attitudes**

**Your personal and professional experience of a whole food plant-based diet.**

**15.As a dietitian, have you personally tried to move to a whole food plant-based diet (i.e. the Eat Lancet Planetary Health Diet: >85% of total energy from whole foods) as mentioned in Section 2?**

**Yes**

**No**

**16. If you answered yes, what food items have you completely excluded/eliminated from your diet? (Multiple answers if applicable).**

**Red meat**

**Poultry**

**Fish**

**Dairy**

**Eggs**

**Honey**

**Not applicable**

**Other**

**17.If you answered No, what are some of the barriers that may impact you from implementing this?**

**Perceived difficulty in following a plant-based diet**

**Not thinking it is a healthy dietary pattern**

**Not getting enough protein or low/poor protein quality**

**Excluding dairy**

**Excluding eggs**

**Financial cost compared to an omnivore diet**

**Concerns around micronutrient deficiencies and having to supplement**

**Challenges in maintaining cultural foods and practices**

**Not applicable**

**Other**

**18.Do you believe that a whole food plant-based diet is a sustainable long-term diet pattern?**

**Strongly agree**

**Agree**

**Not sure**

**Disagree**

**Strongly disagree**

**19.In your specialty, how often would you recommend a whole food plant-based diet to your patients or clients?**

**Never**

**Rarely**

**Sometimes**

**Often**

**Always**

**Not applicable**

**20.Reflecting on this, what are some of the barriers you have come across when implementing a whole food plant-based diet with your patients or clients? (Choose multiple options if applicable)**

**Lack of interest**

**Perceived difficulty in following a plant-based diet**

**Negative beliefs or attitudes in changing to a plant-based diet**

**Concerns around consuming protein exclusively derived from plant origin**

**Excluding certain food groups (dairy/eggs)**

**Financial cost**

**Lack of knowledge about the health benefits of consuming a plant-based diet**

**Food options whilst eating out**

**Preparing meals at home (particularly if the family eating patterns are not the same)**

**Difficulty in changing or adapting ethnic or culturally specific foods from the diet**

**Not applicable**

**Other**

**21.During your dietetic degree, how would you describe the quality of the training and education you received on plant-based nutrition as a therapeutic diet?**

**Very poor**

**Poor**

**Good**

**Very good**

**Excellent**

**Did not receive any training or resources on this topic**

**22.How confident do you feel in counselling a client or patient to transition to a whole food plant-based diet if and when appropriate?**

**Not confident**

**Slightly confident**

**Somewhat confident**

**Fairly confident**

**Completely confident**

**Not applicable**

**Other**

**23.In line with a patient-centred care approach, what preferred dietary approach would you use in order to achieve remission of type 2 diabetes with your clients? (Choose multiple if combining strategies)**

**National dietary guidelines**

**Low carbohydrate diet**

**Ketogenic diet**

**Mediterranean diet**

**DASH diet**

**Vegetarian diet**

**Vegan diet**

**Whole food plant-based diet**

**High protein diet**

**Total dietary replacements (TDR)**

**Partial dietary replacement (PDR)**

**Low energy diet**

**Low fat diet**

**Not applicable**

**Other**

**24.What is your preferred dietary approach for the prevention and management of cardiovascular disease? (Choose multiple if combining strategies)**

**National dietary guidelines**

**Low carbohydrate diet**

**Portfolio dietary pattern**

**Ketogenic diet**

**Mediterranean diet**

**DASH diet**

**HEART UK diet**

**Vegetarian diet**

**Vegan diet**

**Whole food plant-based diet**

**High protein diet**

**Total dietary replacements (TDR)**

**Partial dietary replacement (PDR)**

**Low energy diet**

**Low fat diet**

**Not applicable**

**Other**

**25.What is your preferred dietary approach for the management of weight loss? (Choose multiple if applicable)**

**National dietary guidelines**

**Low carbohydrate diet**

**Portfolio dietary pattern**

**Ketogenic diet**

**Mediterranean diet**

**DASH diet**

**Vegetarian diet**

**Vegan diet**

**Whole food plant-based diet**

**High protein diet**

**Total dietary replacements (TDR)**

**Partial dietary replacement (PDR)**

**Low energy diet**

**Low fat diet**

**Not applicable**

**Other**

**26.What preferred dietary guideline or strategy do you most frequently base your recommendations for cancer prevention? (Choose multiple if applicable)**

**Multiple choice.**

**National Dietary Guidelines**

**World Cancer Research Fund (WCRF) guidelines**

**WHO guidelines on healthy diets**

**American Cancer Society**

**ESPEN cancer dietary guidelines**

**Cancer Research UK**

**Irish Cancer Society**

**Canadian Cancer Society**

**National Cancer Institute**

**Not applicable**

**Other**

**27.Do you feel that a whole food plant-based diet is a realistic diet in terms of financial cost for individuals of lower socioeconomic status?**

**Strongly agree**

**Agree**

**Not sure**

**Disagree**

**Strongly disagree**

**28.As a dietitian, do you feel that there are enough evidence-based educational resources for you and your patients/clients to implement a plant-based diet?**

**Strongly agree**

**Agree**

**Not sure**

**Disagree**

**Strongly disagree**

**Other**

**29.Within a hospital or other healthcare facility setting, a plant-based diet should be an integrated therapeutic diet available for appropriate patients.**

**Strongly agree**

**Agree**

**Not sure**

**Disagree**

**Strongly disagree**

**Other**

**30.Would you feel supported if you were to advocate for a whole food plant-based diet in your workplace?**

**Strongly supported**

**Somewhat supported**

**Neutral**

**Somewhat unsupported**

**Strongly unsupported**

**31.Do you think there is an increased risk of malnutrition (undernutrition without disease as defined by ESPEN terminology) in individuals following a plant-based diet?**

**Strongly agree**

**Agree**

**Not sure**

**Disagree**

**Strongly disagree**

**32.A plant-based diet increases an individuals’ risk in acquiring food-related eating disorders (e.g. Anorexia Nervosa, Bulimia, Binge eating disorder or orthorexia nervosa)?**

**Strongly agree**

**Agree**

**Not sure**

**Disagree**

**Strongly disagree**

**Other**

**33.What is the primary motivation that you have come across in clients switching or considering switching to a plant-based diet?**

**Weight loss**

**To improve their overall health**

**To improve glycaemic control**

**Cardiometabolic health outcomes (e.g. cholesterol, hypertension)**

**Environmental aspect**

**Ethics**

**Not applicable**

**Other**

**34.In your personal and professional opinion, do you feel that clients/patients with chronic health conditions (comorbidities) would adhere to a whole food plant-based diet in the long term?**

**Strongly agree**

**Agree**

**Not sure**

**Disagree**

**Strongly disagree**

**Other**

**35.Please feel free to add any further comments, thoughts or opinions related to the questions you just answered Single line text.**
